# Detachable Modular Blood–Brain Barrier-on-a-Chip with a Concentration Gradient Generator for Stepwise Inflammation Modeling and Drug Evaluation

**DOI:** 10.3390/ph19101655

**Published:** 2026-10-20

**Authors:** Zezhi Li, Haizhu Meng, Xinmeng Liao, Shuqi Wang, Dan Hu, Lijuan Chen, Yuanhang Chang, Junwei Zhao, Xiang Li, Jianping Xie

**Affiliations:** 1 Beijing Life Science Academy, Building 1, Courtyard 7, Yingcai South 1st Street, Changping District, Beijing 102209, China; lzzdyq1992@163.com (Z.L.); liaoxm@blsa.com.cn (X.L.); zhaojw@blsa.com.cn (J.Z.); 2 School of Food and Health, Beijing Technology and Business University, No. 11, Fucheng Road, Haidian District, Beijing 100048, China; wangshuqi@btbu.edu.cn (S.W.); hud@btbu.edu.cn (D.H.); 3 First Clinical Medical College, Shandong Medical and Pharmaceutical University, No. 318, Gangcheng East Street, Laishan District, Yantai 264003, China; mhz9683@163.com; 4 College of Tobacco Science and Engineering, Zhengzhou University of Light Industry, No. 136 Kexue Road, Zhengzhou 450001, China; chenlijuan5210@163.com; 5 College of Pharmacy, Qilu Medical University, No. 1678 Renmin Road, Zibo 255300, China; changyuanhang@qlmu.edu.cn

**Keywords:** blood–brain barrier, organ-on-a-chip, microfluidic concentration gradient, TNF-α, endothelial inflammation, quercetin, kaempferol, ICAM-1

## Abstract

**Background/Objectives**: The blood–brain barrier (BBB) is compromised in many neurological disorders, yet in vitro models rarely reproduce graded inflammatory stimulation of brain endothelial cells in vivo. This study developed a detachable, modular BBB-on-a-chip for stepwise inflammation modeling with multi-marker readout, and compared the anti-inflammatory efficacy of quercetin (QUER) and kaempferol (KAE). **Methods**: The chip combines a Christmas-tree concentration gradient generator with a four-channel culture module, fabricated separately and assembled on demand for stepwise seeding, exposure, dosing, and sampling. hCMEC/D3 cells were exposed to a TNF-α gradient (0–30 ng/mL); extracellular ATP, ROS, NO, cytokines, ZO-1, and ICAM-1 were monitored over 48 h. Transcriptomics and network pharmacology were integrated with molecular docking and 100 ns molecular dynamics simulations; QUER and KAE were administered gradient-wise (0–20 μM), with dexamethasone as a control. **Results**: The TNF-α gradient induced time- and concentration-dependent injury, with ATP and IL-8 responding at 8 h, ROS and NO at 12 h, and IL-1β, IL-6, and tight-junction impairment later, yielding spatiotemporally resolved readouts across four parallel channels. Ten hub inflammatory genes, including *ICAM1*, were identified, and network pharmacology nominated QUER. QUER was predicted to bind ICAM-1 more strongly than KAE, its structural analog differing by one B-ring hydroxyl group; gradient administration confirmed QUER suppressed ICAM-1 expression and inflammatory mediator release at lower concentrations. **Conclusions**: The detachable modular design makes multi-step inflammation modeling and gradient-based anti-inflammatory evaluation convenient on a single chip; QUER’s consistent advantage over KAE across binding calculations and functional assays supports a structure-activity relationship consistent with ICAM-1 engagement.

## 1. Introduction

The blood–brain barrier (BBB) is built upon cerebral microvascular endothelial cells and their intercellular tight junctions and, together with pericytes, astrocytic end-feet, and the basement membrane, forms the neurovascular unit [1]. By strictly regulating the exchange of substances between the blood and the brain parenchyma, it serves as the structural basis for maintaining central nervous system homeostasis [2]. Disruption of this barrier is observed in a range of neurological disorders, including Alzheimer’s disease, ischemic stroke, multiple sclerosis, and traumatic brain injury, and often precedes or accompanies neuronal damage [3,4]. In the onset and progression of neuroinflammation, the blood–brain barrier is not a passive bystander. Pro-inflammatory cytokines typified by TNF-α activate cerebral microvascular endothelial cells, which upregulate adhesion molecules such as ICAM-1 and thereby promote the adhesion and transendothelial migration of circulating leukocytes; meanwhile, tight junctions are disrupted and barrier permeability increases [5,6]. The leaked plasma components and infiltrating immune cells in turn aggravate neuroinflammation, so that barrier damage and inflammatory amplification reinforce each other in a self-perpetuating cycle [7].

To recapitulate this process, a research platform must satisfy at least two requirements at once: it must apply inflammatory stimulation as a controllable concentration gradient, and it must allow candidate compounds to be evaluated in a gradient manner on the same cellular background. Conventional Transwell and multiwell-plate assays can run many samples in parallel, but each insert or well exposes the cells to a single, uniform concentration under static conditions, so that a spatial concentration gradient cannot be imposed on a shared cell population and perfusion is absent; animal models preserve an intact neurovascular unit, yet different doses can only be compared across groups, which likewise precludes continuous dose–response and time–course information [8]. Microfluidic organ-on-a-chip platforms already offer fluid shear stress, dynamic cell culture, and real-time observation [9,10], and gradient generators based on a “Christmas tree” structure can spontaneously form stable concentration gradients within a chip [11,12]. However, most existing designs fix the gradient-generation and cell-culture units into a single monolithic chip, so that cell loading, gradient exposure, drug administration, and sampling must all be performed sequentially on the same device; the numerous tubing connections and fluidic-control steps result in a long operational chain, presenting a steep learning curve for researchers without microfluidic expertise [13].

Alongside these model limitations, effective interventions against inflammatory injury of the cerebrovascular endothelium remain equally scarce. No drug is yet specifically indicated for this process; broad-spectrum anti-inflammatory agents such as glucocorticoids are used mainly for acute-phase control, and their long-term administration is limited by adverse effects including immunosuppression [14]. Natural flavonols such as quercetin (QUER) and kaempferol (KAE) have shown anti-inflammatory and vasoprotective activities in cerebrovascular and other inflammatory models [15,16]. The two compounds differ by only a single hydroxyl group at the 3′ position of the B ring, yet their anti-inflammatory efficacy differs appreciably; whether this disparity stems from differences in their binding to key inflammatory targets remains unsupported by direct molecular evidence [17].

Accordingly, this study developed a detachable, modular, biomimetic BBB chip in which the “Christmas tree” gradient-generation module and the cell-culture module are fabricated separately and assembled on demand, enabling the seeding of human cerebral microvascular endothelial cells, TNF-α gradient exposure, gradient drug administration, and compartment-specific sampling to be performed stepwise on a single platform. After establishing the gradient inflammatory model on the chip and defining its injury patterns, the core inflammatory network was first dissected by transcriptome sequencing. Candidate compounds were then screened through TCMSP-based network pharmacology, and the binding of QUER and KAE to the key target ICAM-1 was compared by molecular docking and molecular dynamics simulations. Finally, gradient drug-administration experiments were conducted to evaluate the effects of both compounds on the release and expression of inflammatory markers and on barrier integrity, with the clinically used drug dexamethasone (DEX) serving as a positive control. Methodologically, the platform contributes a spatiotemporally resolved multi-marker pharmacological readout strategy: four concentration-defined regions are sampled at successive time points for complementary readouts, effluent biomarkers, fluorescence imaging, and transcriptomics, so that the concentration- and time-dependence of inflammatory injury and drug intervention can be resolved on a single platform. This study aims to provide a readily transferable gradient-based strategy for BBB inflammation research and to elucidate, at the level of structure–activity relationships, the molecular basis of QUER’s superior anti-inflammatory efficacy.

## 2. Results

### 2.1. Design and Application Workflow of the Modular Biomimetic Blood–Brain Barrier Chip

**Figure 1:**
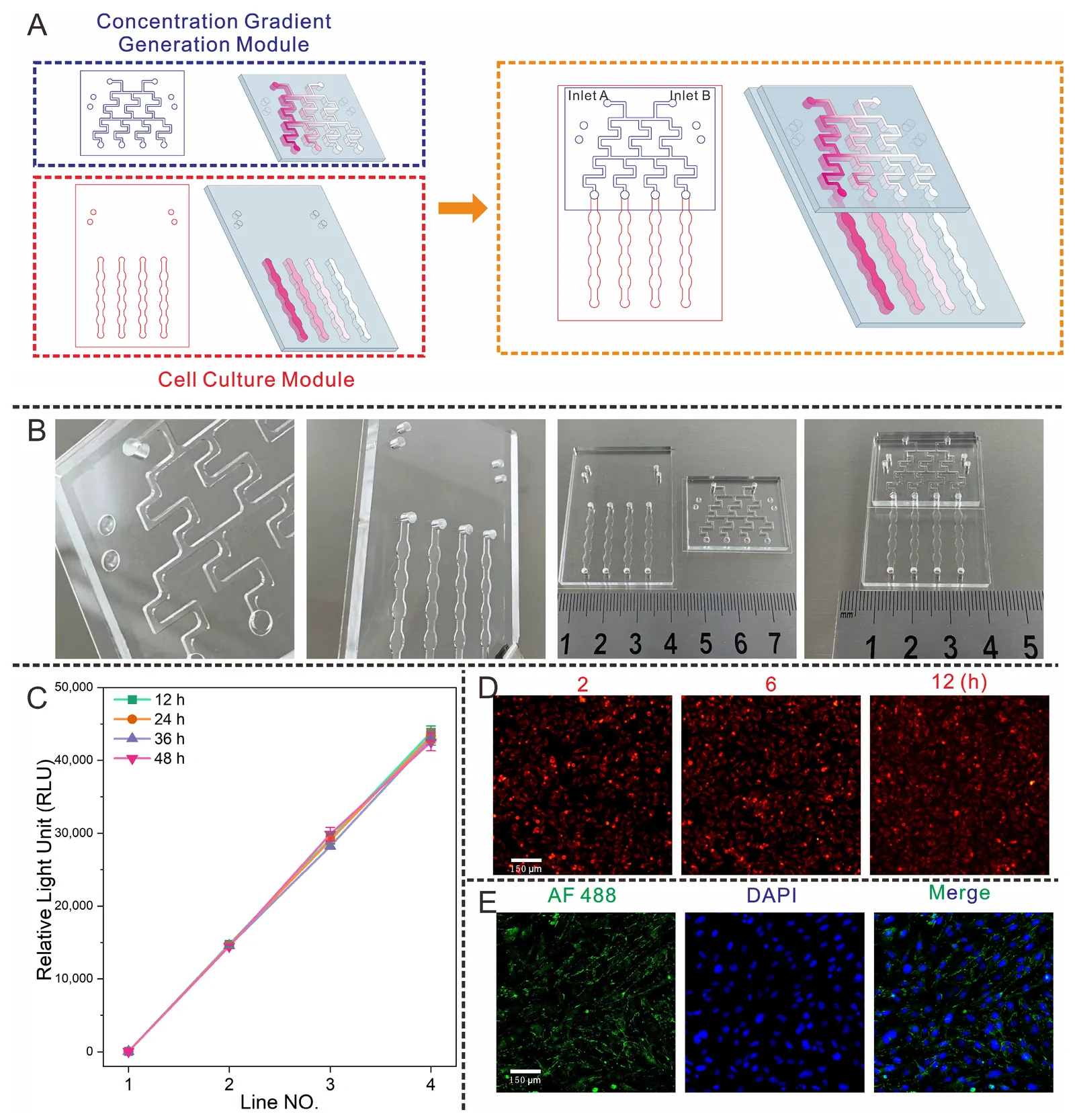
Design, assembly, and functional validation of the detachable modular blood–brain barrier chip. (**A**) Schematic of the chip, comprising a cell culture module with four parallel channels (Line 1–4) and a liquid concentration gradient generator module. (**B**) Photograph of the fabricated chip and close-up views of selected details. (**C**) Characterization of gradient formation using sodium fluorescein as a tracer; fluorescence intensity of the effluent from the four channels was measured every 12 h over 48 h. (**D**) Time-lapse observation of CellTrace^™^ Red CMTPX-labeled hCMEC/D3 cells during 12 h of static culture. (**E**) Immunofluorescence of the tight-junction protein ZO-1 (green) with DAPI nuclear counterstain (blue) after 48 h of dynamic culture. Data in (**C**) are presented as mean ± SD (*n* = 3 independent chips).

As shown in Figure 1A,B, the biomimetic blood–brain barrier (BBB) chip comprises a cell culture module (CCM) and a liquid concentration gradient generator module (LCGGM, Figure S1). Assembly and disassembly of the two modules allow cell seeding, substance exposure or intervention, and sample collection to be performed in separate steps. The center of the CCM contains four parallel fluidic channels that are continuously perfused with medium, keeping the cells adequately supplied with nutrients throughout the experiment. Two alignment holes on each side of the channel region engage the alignment micropillars on the LCGGM, allowing the two modules to be integrated seamlessly. The LCGGM uses a classic Christmas-tree structure that generates a concentration gradient across the four channels by free diffusion, and micropillars flanking the tree enable rapid alignment with the CCM. In the TNF-α exposure and drug-intervention experiments, the LCGGM was attached to the channel inlets of the CCM with pressure-sensitive adhesive. Media containing two different substances were introduced through inlets A and B of the LCGGM, and free diffusion together with stepwise dilution generated a stable concentration gradient (Figure 1A).

### 2.2. Functional Validation of the Chip

Gradient generation was evaluated by stepwise dilution of sodium fluorescein. As shown in Figure 1C, the fluorescence intensity of the effluent was linearly distributed across the four CCM channels and remained stable in each channel throughout the 48 h experiment. To evaluate the biocompatibility and stability of the chip, cells labeled with the red live-cell tracer CellTrace^™^ Red CMTPX were seeded into the CCM channels, cultured statically for 12 h, and observed continuously. The cells began to attach and spread 2 h after seeding and reached confluence by 12 h (Figure 1D). After 48 h of continuous dynamic culture, immunofluorescence staining of the tight-junction protein ZO-1 showed clear enrichment at cell–cell contacts, indicating firm intercellular adhesion and continuous cell–cell junctions (Figure 1E).

### 2.3. Validation of the Inflammation Model

To confirm successful establishment of the gradient inflammation model and to determine the modeling concentration for subsequent drug intervention, effluent from each channel was collected every 8 h during TNF-α exposure and assayed for IL-6, IL-8, IL-1β, and extracellular ATP. These measurements were combined with fluorescence imaging of intracellular reactive oxygen species (ROS) and nitric oxide (NO) and immunofluorescence detection of the tight-junction protein ZO-1, allowing the model to be evaluated at three levels: inflammatory factor secretion, oxidative stress, and barrier integrity. The four culture channels are referred to this work, because the chip was designed from the outset as a multifunctional platform, the contents of the channels differ between experiments, and the condition represented by each line is stated in the corresponding figure caption. In this inflammation-modeling experiment, Lines 1–4 correspond to nominal TNF-α concentrations of 0, 10, 20, and 30 ng/mL, respectively (0%, 33%, 66%, and 100% of the 30 ng/mL inlet concentration).

**Figure 2:**
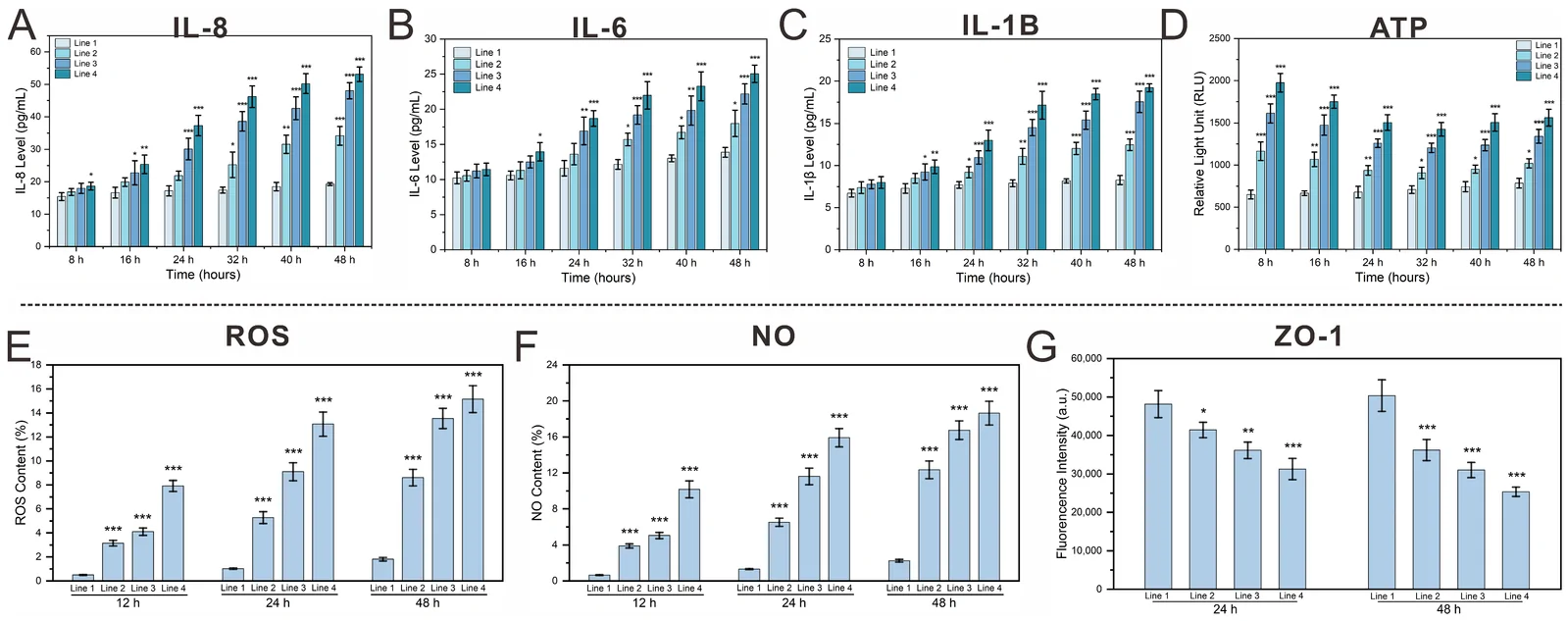
Validation of the gradient inflammation model on the chip. Lines 1–4 received nominal TNF-α concentrations of 0, 10, 20, and 30 ng/mL, respectively (0%, 33%, 66%, and 100% of the inlet concentration), for 48 h. (**A**–**D**) Time courses of IL-8, IL-6, IL-1β, and extracellular ATP in the effluent. (**E**,**F**) Quantification of intracellular ROS and NO fluorescence at 12, 24, and 48 h. (**G**) Quantification of ZO-1 immunofluorescence intensity at 24 and 48 h. Representative fluorescence and immunofluorescence images are provided in Figure S2. Data are presented as mean ± SD (*n* = 3 independent chips). * *p* < 0.05, ** *p* < 0.01, *** *p* < 0.001 vs. Line 1.

Dynamic monitoring of the three cytokines and extracellular ATP revealed a clear temporal division of labor (Figure 2A–D). Line 1 (0 ng/mL) maintained only low basal secretion throughout the observation period, whereas Lines 2–4 were activated in a concentration-dependent manner. Among the cytokines, IL-8 responded fastest and with the largest amplitude: Lines 3 and 4 (20 and 30 ng/mL) were already significantly higher than Line 1 at 8 h, Line 2 (10 ng/mL) rose significantly from 16 h onward, and a concentration-ordered gradient was still maintained at 48 h. IL-1β and IL-6 responded more slowly, exceeding Line 1 in succession from 16 h and then accumulating in a time- and concentration-dependent manner, with IL-6 showing the latest onset and the gentlest rise. Extracellular ATP followed a distinctly different, early-release pattern: at 8 h it had already risen sharply across Lines 2–4 with a clear concentration gradient, the earliest response among all the indicators measured. ATP levels in Lines 2–4 then declined gradually, and although the gap with Line 1 narrowed over time, it remained significant through 48 h.

Fluorescence imaging of ROS and NO showed dependence on both time and concentration (Figure 2E,F; representative images in Figure S2A,B). At 12 h of exposure, the signals in Lines 2–4 were already significantly stronger than in Line 1; they continued to rise thereafter while maintaining a gradient that intensified with concentration. By 48 h, ROS and NO levels in Lines 3 and 4 had converged, suggesting that the oxidative-stress response was approaching saturation, whereas Line 1 showed only minimal fluorescence throughout. ZO-1 immunofluorescence corroborated this damage at the level of barrier structure (Figure 2G; representative images in Figure S2C). In Line 1, ZO-1 was distributed as a continuous line along cell–cell contacts at both detection time points. In Lines 2–4, ZO-1 fluorescence decreased in a graded manner with increasing concentration, and the staining pattern shifted from continuous lines to a discontinuous, blurred distribution; the damage was more severe at 48 h than at 24 h, indicating that gradient TNF-α exposure progressively disrupts tight junctions between endothelial cells.

Taken together, these results indicate that on-chip TNF-α exposure induced a stable and sustainable graded inflammatory response: pro-inflammatory cytokines were released cumulatively over time, extracellular ATP was released rapidly and in a graded manner at the early stage of exposure, ROS and NO were activated early, and ZO-1 was down-regulated along the concentration gradient. The gradient BBB inflammation model was thus successfully established. Because 100% TNF-α (30 ng/mL) induced the strongest inflammatory response with good within-group reproducibility, it was selected as the modeling concentration for the subsequent drug-intervention experiments.

### 2.4. Differential Gene Expression Profiling After TNF-α Exposure

**Figure 3:**
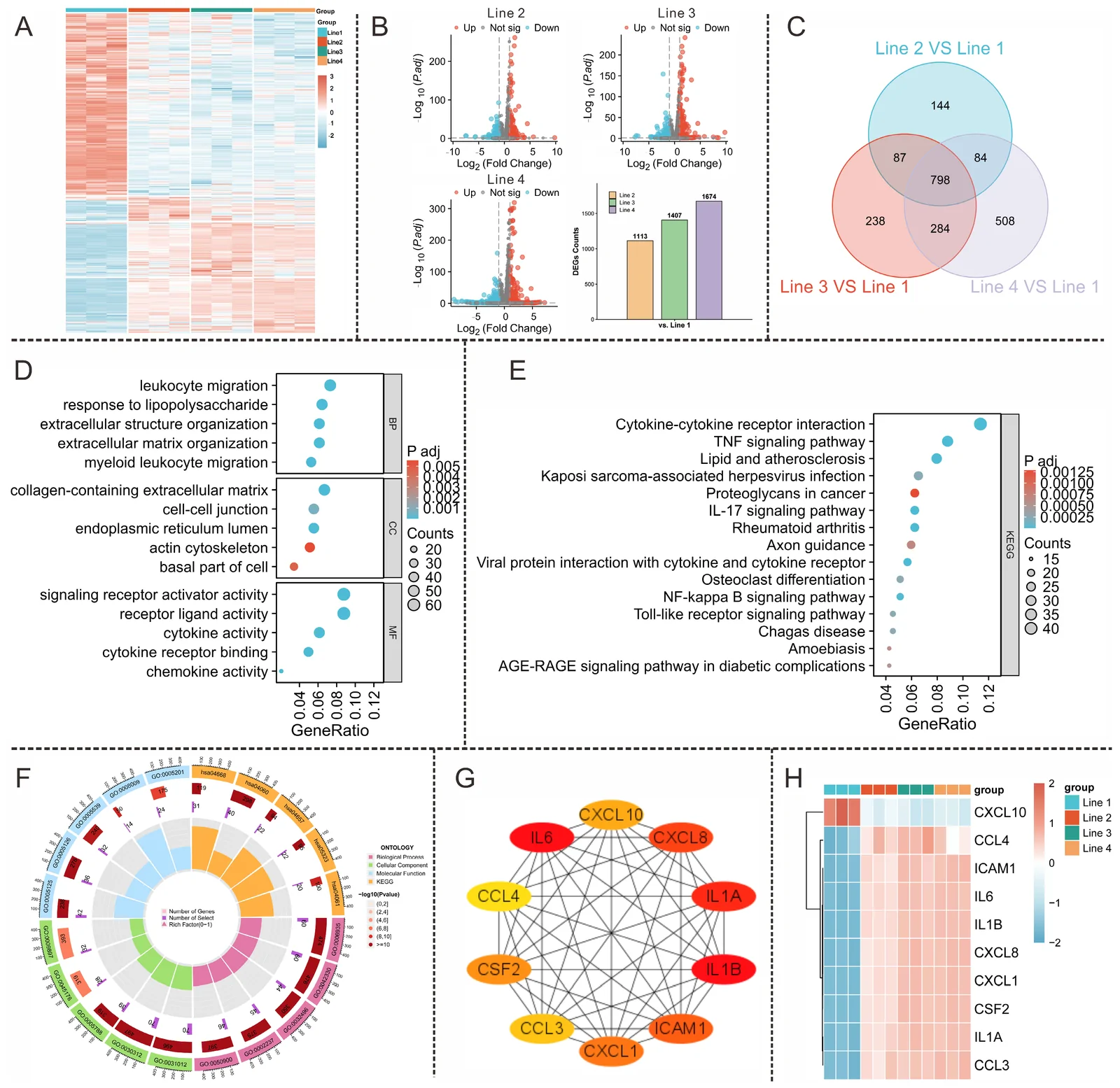
Transcriptomic analysis of hCMEC/D3 cells under TNF-α gradient exposure. (**A**) Genome-wide expression heatmap of all samples. (**B**) Numbers of DEGs in Lines 2–4 (10, 20, and 30 ng/mL) relative to Line 1 (0 ng/mL) (|log2FC| > 0.5, adjusted *p* < 0.05). (**C**) Venn diagram of DEGs across the three comparisons. (**D**) GO enrichment analysis. (**E**) KEGG pathway enrichment analysis. (**F**) Circular plot integrating the enrichment results. (**G**) Ten hub genes identified by maximal clique centrality (MCC) topological analysis, together with their expression heatmap. (**H**) Protein–protein interaction (PPI) network of DEGs from inflammation-related pathways.

With the model validated, the transcriptional response to gradient TNF-α exposure was examined next. The transcriptional response of hCMEC/D3 cells to inflammatory cytokines has been mapped previously [18]; transcriptomics was therefore used here to confirm that the on-chip gradient reproduces this established program in a concentration-resolved manner and to nominate the effector nodes carried forward for target analysis. The genome-wide expression heatmap (Figure 3A) showed that all samples clustered clearly by experimental group, with good agreement among within-group replicates; Line 1 (0 ng/mL) was clearly separated from the treated lines (Lines 2–4), and the deviation of the expression profiles widened progressively with increasing TNF-α concentration, reflecting continuous dose-dependent remodeling.

Differentially expressed genes (DEGs) were screened against Line 1 (0 ng/mL) (|log2FC| > 0.5, adjusted *p* < 0.05). A total of 1113, 1407, and 1674 DEGs were identified in Lines 2, 3, and 4 (10, 20, and 30 ng/mL), respectively (Figure 3B). The total number of DEGs increased steadily with concentration, indicating that stronger stimulation remodels the transcriptional program more extensively.

The Venn diagram (Figure 3C) showed 798 DEGs shared by all three groups, constituting a stable gene set underlying the TNF-α inflammatory response, whereas the group-specific DEGs (144, 238, and 508, respectively) increased markedly with TNF-α concentration. GO annotation (Figure 3D) showed that, at the biological-process level, the DEGs were mainly enriched in inflammation- and immunity-related terms such as chemotaxis, response to lipopolysaccharide and molecules of bacterial origin, and leukocyte migration; at the cellular-component level, the enriched terms concentrated in the extracellular matrix, the endoplasmic reticulum lumen, the basal part of the cell, and the external side of the plasma membrane; and at the molecular-function level, cytokine activity, cytokine receptor binding, and glycosaminoglycan binding predominated. The top-ranked KEGG pathways (Figure 3E) were the TNF signaling pathway, cytokine–cytokine receptor interaction, and the IL-17 signaling pathway, together with other inflammation-related pathways such as NF-κB and Toll-like receptor signaling. The circular plot integrating these enrichment results (Figure 3F) showed a landscape dominated by biological-process terms, with cytokine- and chemokine-related pathways highly clustered and several core inflammatory pathways reaching very high levels of significance.

DEGs from inflammation-related pathways, including cytokine–cytokine receptor interaction, the IL-17 signaling pathway, and the NF-κB signaling pathway, were pooled and mapped to the STRING database to construct a protein–protein interaction network. MCC topological analysis identified 10 core hub genes (Figure 3G,H): *IL6*, *IL1B*, *CXCL8*, *CXCL1*, *CSF2*, *ICAM1*, *CXCL10*, *CCL3*, *CCL4*, and *IL1A*, all established mediators of endothelial inflammatory activation, with interleukin and chemokine family members forming the core nodes of the network. The hub-gene expression heatmap showed that, with the exception of *CXCL10*, which was progressively down-regulated with increasing TNF-α concentration, the remaining nine genes were all up-regulated along the gradient, corroborating at the transcriptome level the successful establishment of the gradient inflammation model.

### 2.5. Validation of the Transcriptomic Results

To verify the reliability of the transcriptome sequencing results, five representative hub genes from the PPI network (*IL6*, *IL1B*, *IL1A*, *CXCL8*, and *ICAM1*) were selected for RT-qPCR analysis. Given that the secreted protein levels of IL-6, IL-8, and IL-1β had already been validated in the effluent assays (Section 2.3), only ICAM-1 expression was additionally examined at the protein level by immunofluorescence.

**Figure 4:**
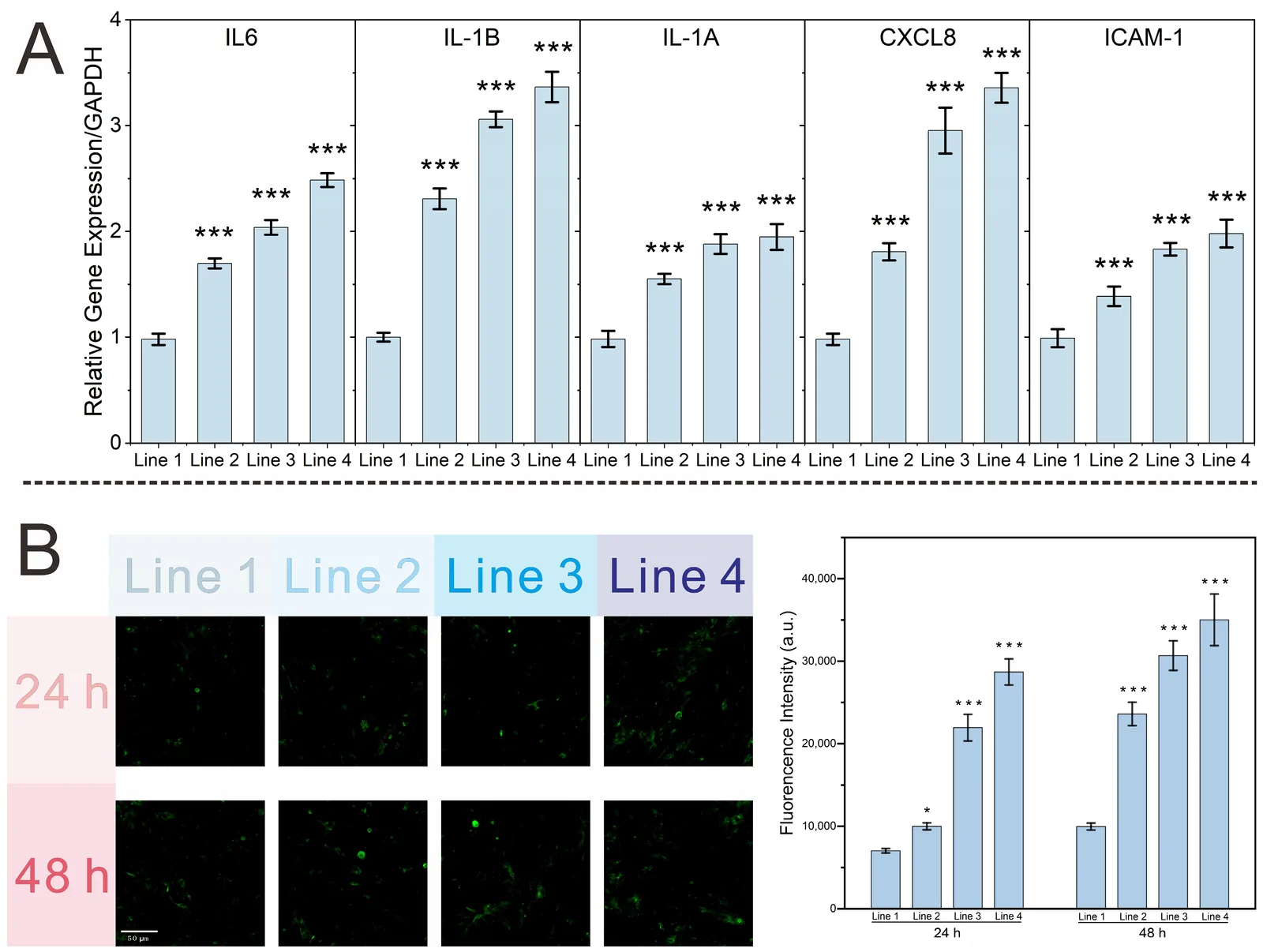
Validation of the transcriptomic results. (**A**) RT-qPCR analysis of the five hub genes (*IL6*, *IL1B*, *IL1A*, *CXCL8*, and *ICAM1*) at 48 h. (**B**) ICAM-1 immunofluorescence (green) at 24 and 48 h. Data are presented as mean ± SD (*n* = 3 independent chips). * *p* < 0.05, *** *p* < 0.001 vs. Line 1.

The RT-qPCR results (Figure 4A) showed that the relative expression of all five genes was significantly higher in every treated line (Lines 2–4) than in Line 1 and increased concentration-dependently with TNF-α exposure. *IL1B* and *CXCL8* showed the largest up-regulation, whereas *IL1A* and *ICAM1* rose more moderately and leveled off between Lines 3 and 4 (20 and 30 ng/mL), a pattern consistent with the transcriptomic heatmap; the expression trends of the individual genes agreed well with the sequencing data. ICAM-1 immunofluorescence (Figure 4B) showed that, at 24 h of exposure, the fluorescence intensity in Lines 2–4 was already significantly higher than in Line 1 and increased with TNF-α concentration, and the differences remained highly significant at 48 h. The concordance of RT-qPCR and immunofluorescence with the transcriptome sequencing results at both the mRNA and protein levels indicates that the up-regulation of the core inflammatory genes induced by gradient TNF-α exposure is reliable.

### 2.6. Molecular Docking and Molecular Dynamics Simulations of Quercetin and Kaempferol with ICAM-1

**Figure 5:**
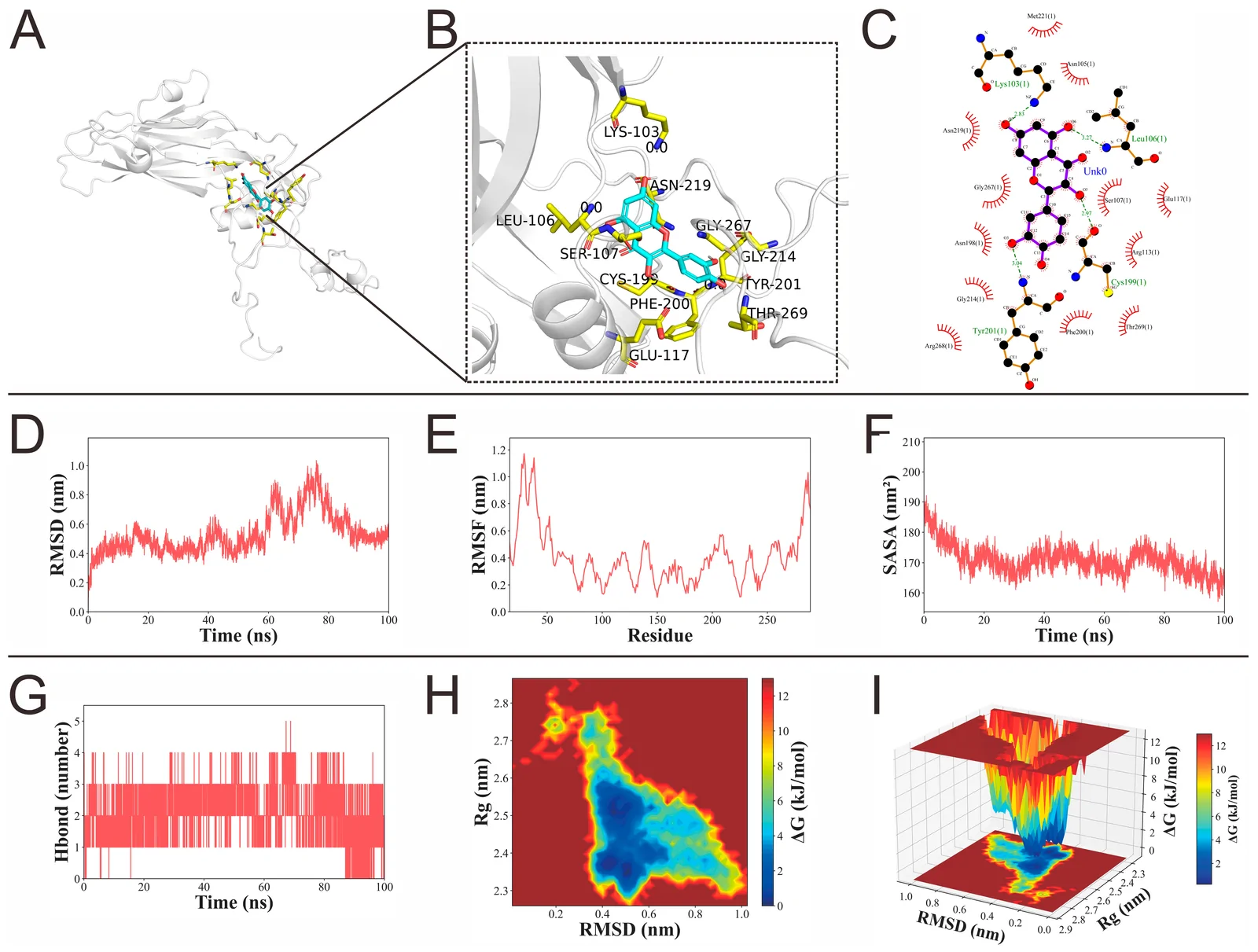
Molecular docking and 100 ns molecular dynamics (MD) simulation of the QUER–ICAM-1 complex. (**A**–**C**) Binding mode of QUER within the ICAM-1 pocket: overall view (**A**), close-up of hydrogen-bonding residues (**B**), and two-dimensional interaction diagram (**C**). (**D**) Root-mean-square deviation (RMSD) of the complex. (**E**) Root-mean-square fluctuation (RMSF) of protein residues. (**F**) Solvent-accessible surface area. (**G**) Number of protein–ligand hydrogen bonds over time. (**H**,**I**) Gibbs free energy landscape of the complex.

**Figure 6:**
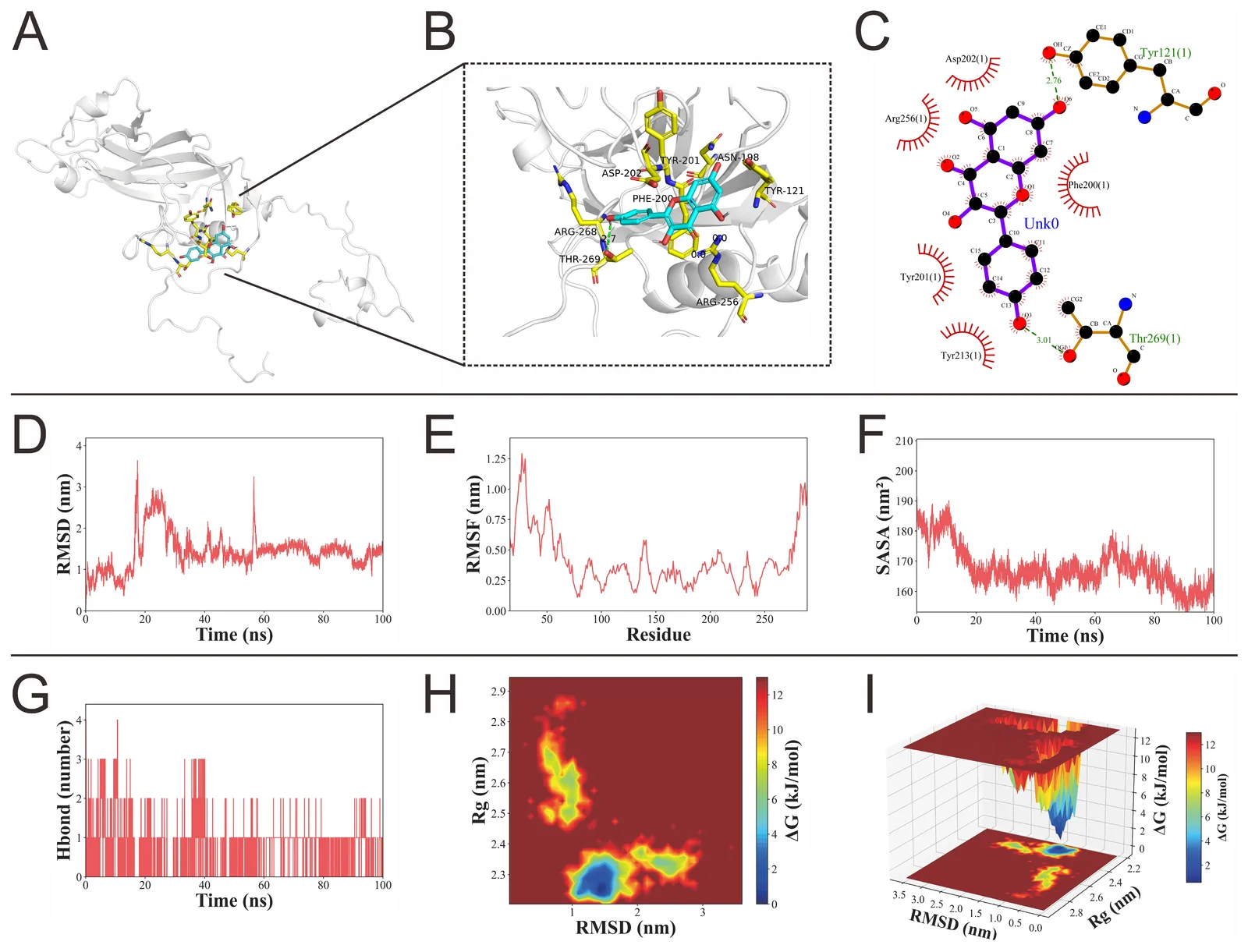
Molecular docking and 100 ns molecular dynamics (MD) simulation of the KAE–ICAM-1 complex. (**A**–**C**) Binding mode of KAE within the ICAM-1 pocket: overall view (**A**), close-up of hydrogen-bonding residues (**B**), and two-dimensional interaction diagram (**C**). (**D**) RMSD of the complex. (**E**) RMSF of protein residues. (**F**) Solvent-accessible surface area. (**G**) Number of protein–ligand hydrogen bonds over time. (**H**,**I**) Gibbs free energy landscape of the complex.

The above results suggested that the inflammatory network composed of IL-6, IL-1β, IL-8 (*CXCL8*), and ICAM-1 is central to TNF-α-induced BBB injury. We therefore used these four molecules as targets, retrieved their related ingredients from the TCMSP database, and took the intersection to screen for candidate compounds capable of acting on multiple targets; the intersecting ingredients pointed to QUER. KAE belongs to the same flavonol subclass as QUER, differs structurally only by a single hydroxyl group at the 3′ position of the B ring, and has also been reported to possess anti-inflammatory activity; the two compounds thus constitute a natural pair for structure–activity comparison. Because ICAM-1 is the membrane protein in the hub network that directly mediates leukocyte adhesion, the two flavonoids were docked separately against ICAM-1, and the resulting complexes were subjected to 100 ns molecular dynamics (MD) simulations. The docking results (Figure 5 and Figure 6) showed that the binding energy of QUER to ICAM-1 was −8.14 kcal/mol, clearly more favorable than that of KAE (−6.67 kcal/mol).

Binding-mode analysis showed that QUER inserts into the binding pocket of ICAM-1 and forms four hydrogen bonds with Lys103, Leu106, Cys199, and Tyr201, with donor–acceptor distances of 2.83–3.27 Å, together with extensive hydrophobic interactions involving Asn105, Ser107, Arg113, Glu117, Asn198, Phe200, Gly214, Asn219, Met221, Gly267, and Arg268 (Figure 5A–C). KAE formed only two hydrogen bonds, with Tyr121 and Thr269 at distances of 2.76 and 3.01 Å, respectively, and its hydrophobic contacts were limited to Phe200, Tyr201, Asp202, Tyr213, and Arg256 (Figure 6A–C). The two binding regions partially overlap, but QUER achieves stronger binding through more hydrogen bonds and a broader contact network, consistent with the additional hydrogen-bond donor provided by the ortho-dihydroxyl groups on its B ring.

MD simulations were then used to examine the stability of the two complexes in a dynamic environment (Figure 5 and Figure 6). Root-mean-square deviation (RMSD) analysis showed that the QUER–ICAM-1 complex fluctuated within a limited range throughout the simulation, remaining mostly between 0.3 and 0.6 nm; only a brief conformational fluctuation occurred at approximately 70–85 ns, after which the complex settled back and returned to its initial level toward the end of the trajectory (Figure 5D). The KAE–ICAM-1 complex deviated markedly from its initial conformation at the very beginning of the simulation: a transient large fluctuation appeared at around 17 ns, the RMSD stayed within a high range of 2.0–2.9 nm between 20 and 30 ns, spiked briefly again at approximately 56 ns, and then fluctuated mainly between 1.0 and 1.8 nm over 60–100 ns, indicating that the complex reached dynamic equilibrium only after substantial conformational rearrangement (Figure 6D). The root-mean-square fluctuation (RMSF) profiles of the two systems were similar in shape: pronounced fluctuations were concentrated in the N- and C-termini and in a few loop regions, whereas residues near the binding pocket fluctuated little (Figure 5E and Figure 6E). Solvent-accessible surface area (SASA) decreased slightly at the beginning of the simulations and then remained stable (Figure 5F and Figure 6F), indicating that the overall structure of ICAM-1 was well maintained in both systems and that the RMSD differences arose mainly from the different ligand-binding states. Hydrogen-bond analysis showed that QUER maintained one to three hydrogen bonds with the protein throughout the simulation (Figure 5G), whereas KAE maintained only zero to two for most of the trajectory, accompanied by dynamic breakage and reformation (Figure 6G). The Gibbs free energy landscape of the QUER–ICAM-1 complex displayed a single pronounced global basin corresponding to a dominant conformation close to the initial docked pose (Figure 5H,I). For the KAE–ICAM-1 complex, the main basin was located 1.2–1.6 nm away from the initial conformation, with secondary distributions extending to even larger deviations, indicating that its dominant conformation drifted from the docked pose and that multiple metastable states exist (Figure 6H,I).

Combining the docking and simulation results, the QUER–ICAM-1 complex forms more hydrogen bonds, maintains them more persistently, and adopts a more stable binding conformation, whereas the KAE complex forms fewer hydrogen bonds and undergoes pronounced conformational rearrangement. These differences explain, at the atomic level, the gap in binding energy between the two flavonoids, parallel their different anti-inflammatory activities, and are consistent with the hypothesis that ICAM-1 engagement contributes to the anti-inflammatory action of QUER, a possibility that requires experimental confirmation.

### 2.7. Effects of Quercetin and Kaempferol on the Release and Expression of Inflammation-Related Biomarkers

Having determined the optimal modeling concentration and the release pattern of inflammatory markers, we next used the gradient-generation function of the chip, with the clinically used anti-inflammatory drug DEX as a positive control, to create simultaneous drug gradients of 0, 6.67, 13.33, and 20 μM under constant 30 ng/mL TNF-α exposure. QUER and KAE were evaluated in parallel for their effects on the release and expression of inflammation-related biomarkers, using the same readouts as in Section 2.3 and Section 2.5.

**Figure 7:**
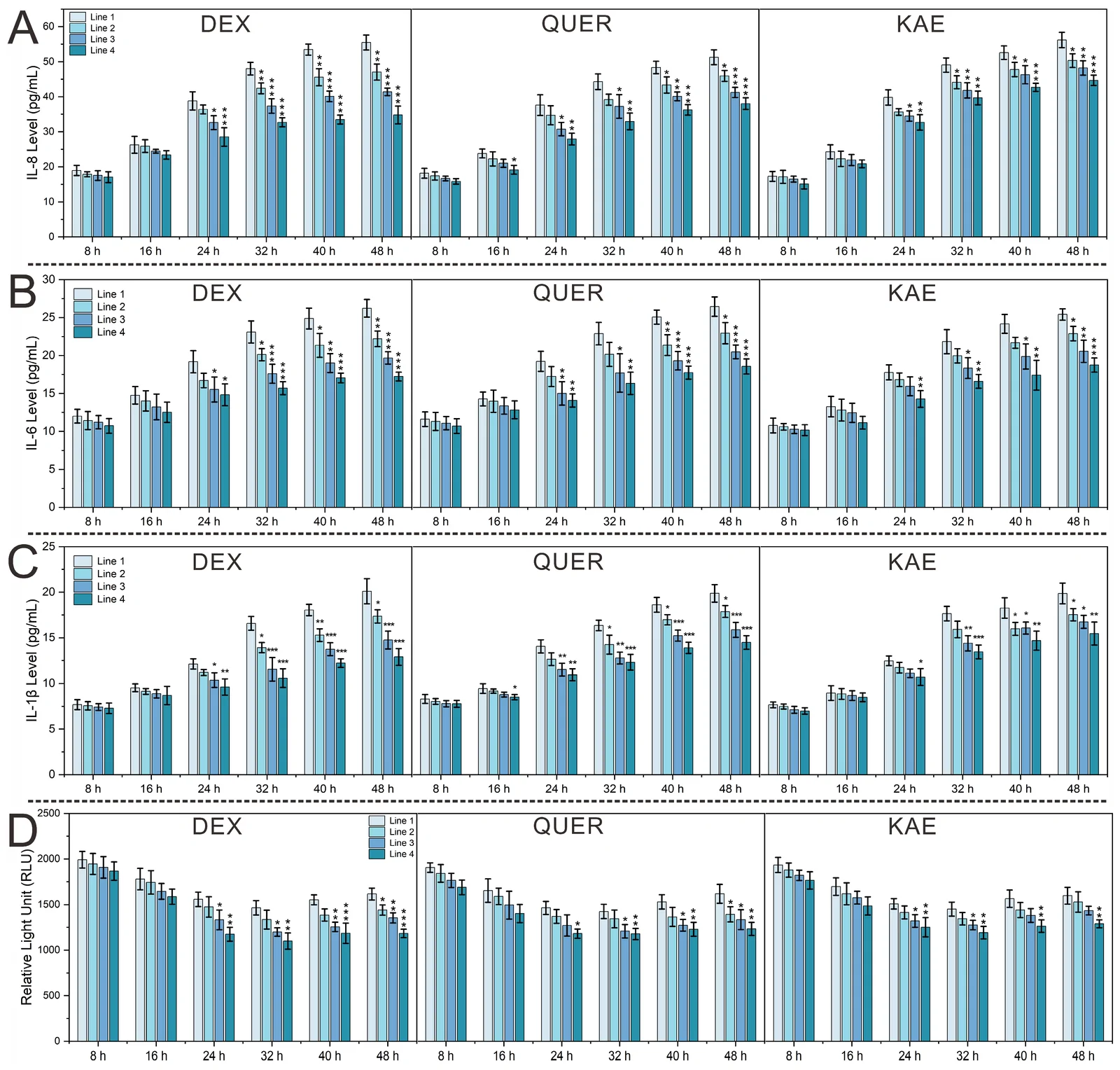
Effects of the drug concentration gradients on cytokine secretion and extracellular ATP release. Under constant 30 ng/mL TNF-α exposure, Lines 1–4 received nominal drug concentrations of 0, 6.67, 13.33, and 20 μM DEX, QUER, or KAE, respectively, for 48 h. (**A**–**C**) Time courses of IL-8, IL-6, and IL-1β in the effluent, collected every 8 h. (**D**) Extracellular ATP levels. Data are presented as mean ± SD (*n* = 3 independent chips). * *p* < 0.05, ** *p* < 0.01, *** *p* < 0.001 vs. the injury control (0 μM).

Dynamic monitoring of the three cytokines (Figure 7A–C) showed that, at the early stage of intervention (8 h), none of the compound-treated groups differed significantly from the injury control. Significant inhibition of IL-8 secretion by QUER appeared as early as 16 h (at 13.33 and 20 μM), whereas the effect of DEX did not reach significance until 24 h. Thereafter, all three compounds reduced the secretion of the three cytokines dose-dependently, and the inhibition strengthened over time; by 48 h, the 13.33 and 20 μM groups of each compound were significantly lower than the injury control. At the same concentration, DEX produced the greatest inhibition, followed by QUER, with KAE being relatively mild. Extracellular ATP followed a similar pattern, with all three compounds dose-dependently lowering ATP levels in the culture medium (Figure 7D). At 20 μM, QUER significantly reduced ATP release as early as 8 h after administration (*p* < 0.05), earlier than DEX and KAE; from 24 h onward, the inhibitory effects of all three compounds remained significant through 48 h. At 48 h, DEX was significant even at the low concentration of 6.67 μM, QUER was significant at 13.33 μM, and KAE reached statistical significance only at 20 μM.

**Figure 8:**
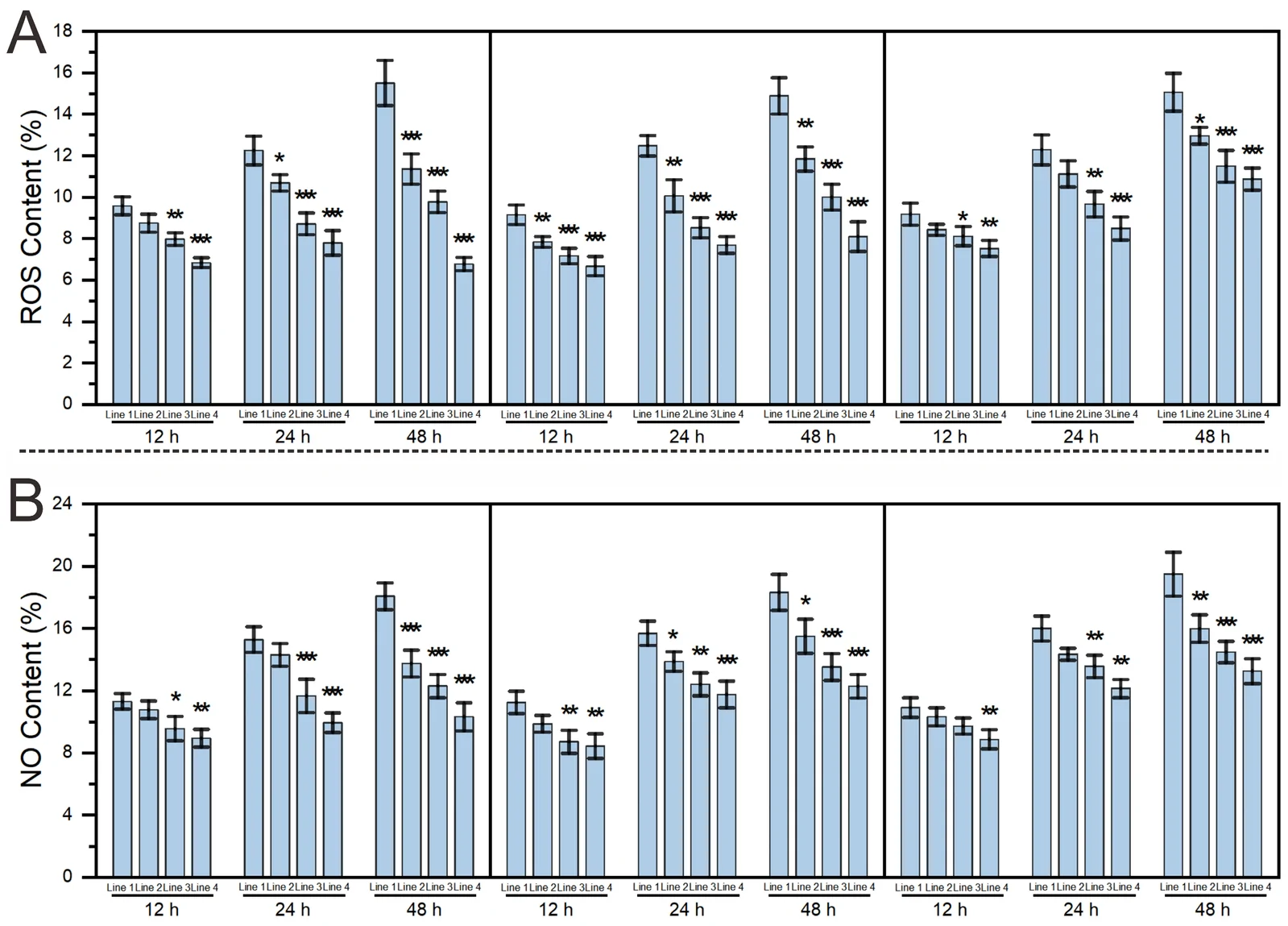
Effects of the drug concentration gradients on intracellular ROS (**A**) and NO (**B**) levels. Lines 1–4 received nominal drug concentrations of 0, 6.67, 13.33, and 20 μM DEX, QUER, or KAE, respectively, under constant 30 ng/mL TNF-α exposure. Fluorescence intensities were quantified at 12, 24, and 48 h; representative images are provided in Figure S3. Data are presented as mean ± SD (*n* = 3 independent chips). * *p* < 0.05, ** *p* < 0.01, *** *p* < 0.001 vs. the injury control (0 μM).

Fluorescence imaging of ROS and NO showed similar dose-dependent trends (Figure 8; representative images in Figure S3). At 12 h, QUER had already significantly lowered ROS at 6.67 μM, and the 20 μM KAE group was also significantly reduced, whereas DEX required concentrations of 13.33 μM or higher to produce a significant difference. By 48 h, ROS levels in the 20 μM groups of all three compounds were significantly lower than in the injury control, and NO levels were significantly reduced as well.

**Figure 9:**
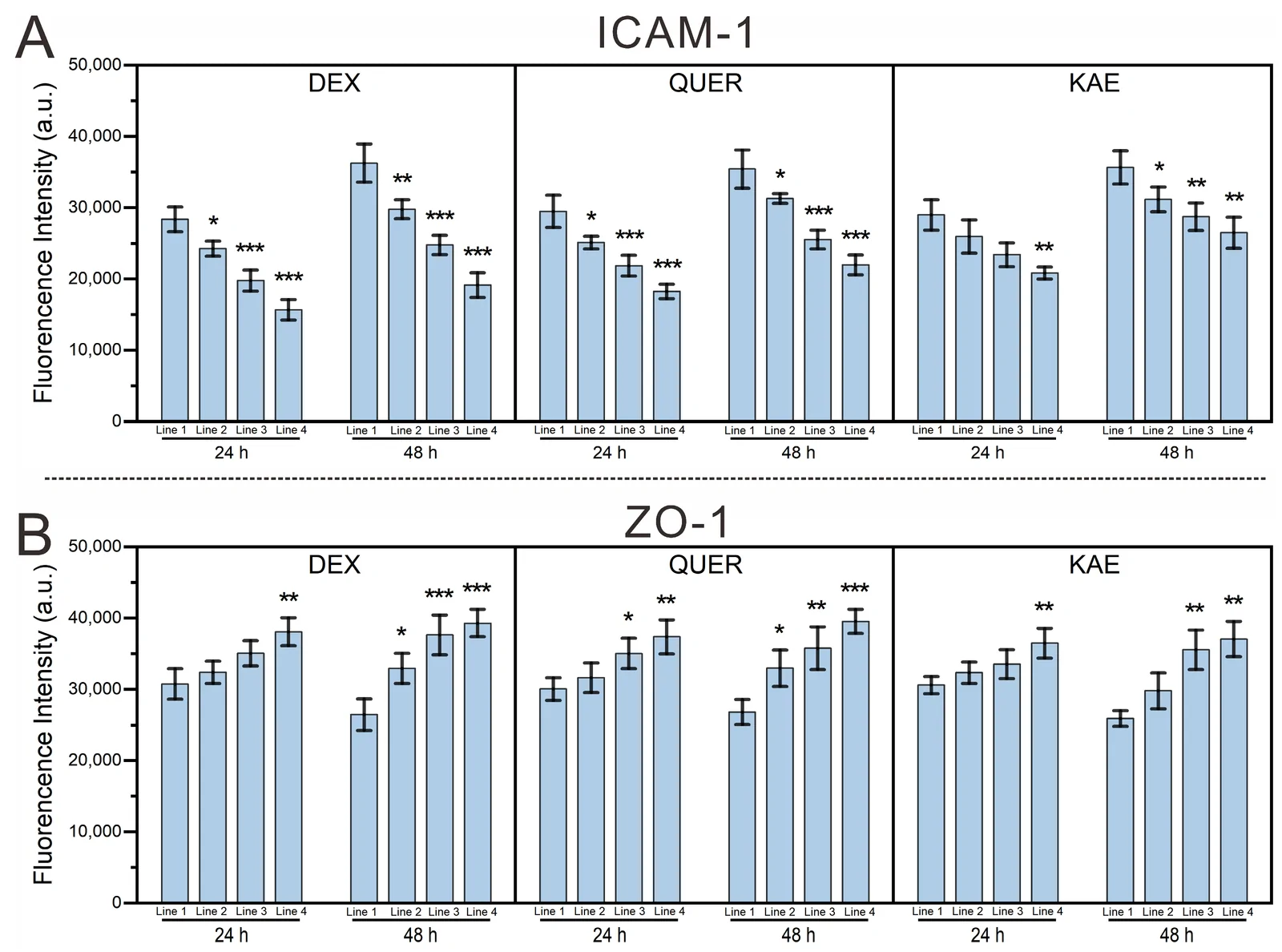
Effects of the drug concentration gradients on ICAM-1 and ZO-1 expression. Lines 1–4 received nominal drug concentrations of 0, 6.67, 13.33, and 20 μM DEX, QUER, or KAE, respectively, under constant 30 ng/mL TNF-α exposure. (**A**) Quantification of ICAM-1 immunofluorescence intensity at 24 and 48 h. (**B**) Quantification of ZO-1 immunofluorescence intensity at 24 and 48 h. Representative immunofluorescence images are provided in Figure S4. Data are presented as mean ± SD (*n* = 3 independent chips). * *p* < 0.05, ** *p* < 0.01, *** *p* < 0.001 vs. the injury control (0 μM).

The effects of the three compounds on ICAM-1 expression are shown in Figure 9A (Figure S4A). ICAM-1 fluorescence intensity increased significantly in endothelial cells after TNF-α injury, and all three compounds reduced it dose-dependently. DEX and QUER significantly down-regulated ICAM-1 from 6.67 μM onward (*p* < 0.05), with stronger inhibition at higher concentrations. KAE required higher concentrations to take effect: it did not differ significantly from the injury control at 6.67 μM, and significant inhibition appeared only from 13.33 μM, a pattern that held at both 24 and 48 h. Between-group comparison at 20 μM showed that the inhibition of ICAM-1 by QUER was significantly stronger than that by KAE at 48 h (*p* < 0.05). In contrast, all three compounds dose-dependently attenuated the loss of ZO-1 expression (Figure 9B and Figure S4B). At 24 h, only the high-concentration groups showed significantly alleviated ZO-1 down-regulation; at 48 h, the 13.33 and 20 μM groups were all significantly higher than the injury control, and the images showed markedly less interruption and fragmentation of ZO-1 at cell–cell contacts in the treated groups, with the continuous line-like distribution preserved. At 48 h, the protective effect of 20 μM QUER was comparable to that of DEX.

**Figure 10:**
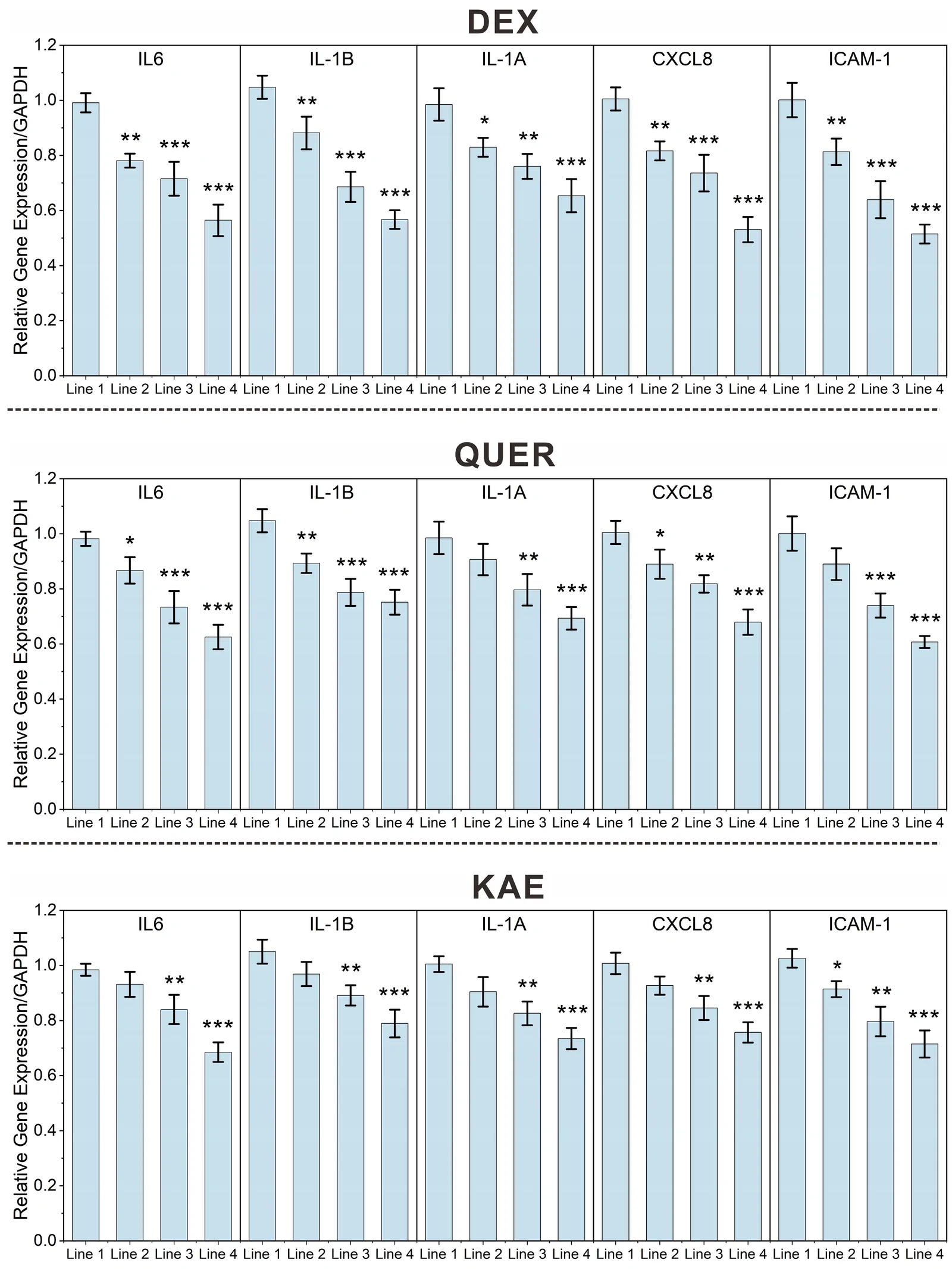
Effects of the drug concentration gradients on hub-gene transcription. RT-qPCR analysis of the five hub genes (*CXCL8*, *IL1B*, *IL6*, *IL1A*, and *ICAM1*) at 48 h. Data are presented as mean ± SD (*n* = 3 independent chips). * *p* < 0.05, ** *p* < 0.01, *** *p* < 0.001 vs. the injury control (0 μM).

The effects of the three compounds on hub-gene expression were further examined at the transcriptional level (Figure 10). Calibrated against the 0 μM injury control, RT-qPCR showed that, after 48 h of treatment, all three compounds dose-dependently down-regulated the mRNA expression of *CXCL8*, *IL1B*, *IL6*, *IL1A*, and *ICAM1*, consistent in direction with the changes in cytokine release and ICAM-1 protein. With DEX, the down-regulation of all five genes was already significant at 6.67 μM (*p* < 0.05). QUER significantly down-regulated *CXCL8* and *IL1B* at 6.67 μM and significantly inhibited all five genes from 13.33 μM onward. For KAE, only the down-regulation of *ICAM1* approached significance at 6.67 μM, and all genes were significantly reduced from 13.33 μM. At 20 μM, QUER inhibited *ICAM1* mRNA significantly more strongly than KAE, echoing the between-group difference at the ICAM-1 protein level, and its inhibition of *IL6* and *IL1A* approached that of DEX.

In summary, QUER produced clear dose-dependent inhibition of the TNF-α-induced inflammatory response, with an overall effect superior to that of KAE; the advantages were most evident for ROS production and ICAM-1 expression. Its attenuation of ZO-1 loss was comparable to that of DEX, and it acted earlier on cytokine release, ATP, and ROS. These findings are consistent with the structure–activity trend suggested by molecular docking and MD simulations.

## 3. Discussion

The blood–brain barrier (BBB) is the critical interface for maintaining central nervous system homeostasis, and its dysfunction is closely associated with neurological disorders such as Alzheimer’s disease and ischemic stroke [3]. During neuroinflammation, pro-inflammatory cytokines such as TNF-α amplify inflammatory signaling while also directly damaging the tight junctions of brain microvascular endothelial cells, up-regulating adhesion molecules, and recruiting leukocytes to migrate across the endothelium, so that the BBB changes from a victim of inflammation into a participant in the inflammatory cascade [3,19]. This response is, however, an inherently concentration-dependent and dynamic process. As noted above, chips that combine gradient generation with cell culture do exist, but their one-piece structure forces cell loading, exposure, drug administration, and sampling to be completed in succession on a single chip, which is demanding for researchers without an engineering background [3,8,10]. We therefore fabricated the Christmas-tree concentration gradient generator module and the cell culture module as separate parts to be assembled on demand, and built a detachable, modular, biomimetic BBB chip. The modular design allows gradient exposure, gradient intervention, and region-specific sampling to be performed stepwise on the same platform, and the resulting stable concentration gradient serves both to optimize injury conditions and to feed directly into drug-dose screening. On this basis, we integrated transcriptomics, network pharmacology, molecular docking, molecular dynamics simulation, and multi-time-point functional assays to verify the candidate ingredient layer by layer, from the gene network down to molecular binding. QUER and KAE differ by only one hydroxyl group, and comparing them provides a natural control for resolving the structural origin of flavonoid anti-inflammatory potency. This strategy is not confined to the present subject; it applies equally to the screening and mechanistic analysis of other natural-product ingredients and may inform a range of research needs [17]. Following this strategy, we first established a stable and reproducible TNF-α-gradient model of BBB inflammation on the chip and proceeded from there. Transcriptome sequencing pinpointed a hub inflammatory network centered on *IL6*, *IL1B*, *CXCL8*, and *ICAM1*; TCMSP screening accordingly pointed to QUER as the candidate ingredient; molecular docking and dynamics simulations further predicted that QUER binds ICAM-1 more favorably than its structural analog KAE; and gradient dosing experiments ultimately confirmed this prediction, namely that QUER shows stronger overall anti-inflammatory efficacy than KAE, delays ZO-1 damage to a degree comparable to DEX, and acts earlier on several readouts.

The inflammatory injury of brain microvascular endothelium by TNF-α is itself well documented, and we do not present it as a new finding. TNF-α downregulates occludin and increases cerebral endothelial permeability through p38MAPK signaling [5]; in hCMEC/D3 cells specifically, TNF-α induces MMP-9 through the Ca^2+^/CaMKII/ERK/NF-κB pathway and impairs barrier integrity [6]; cytokine exposure remodels the hCMEC/D3 transcriptome along the same cytokine, IL-17, and NF-κB pathways recovered in our data [18]; and, on chip, TNF-α has been shown to reduce transendothelial electrical resistance in an hCMEC/D3-based microfluidic BBB model [9]. The present results are fully consistent with this literature and extend it only along the concentration axis: the platform reads out this established program simultaneously at four concentrations on a shared cell background and couples it to gradient drug evaluation on the same device.

The chip platform was designed around two requirements: stepwise operation and gradient generation. Unlike existing designs that fix the gradient unit and the culture chamber together as one piece, the cell culture module and the gradient generator module in this study are reversibly integrated through alignment holes and micropillars. At the seeding stage, the standalone culture module allows the cell suspension to be perfused directly and to spread quickly and evenly. At the injury-induction and dosing stages, assembling the gradient module immediately yields four parallel concentrations. At the detection stage, the modules can be separated so that effluent and cells are collected channel by channel and fluorescence staining is completed. ZO-1 showed a continuous linear distribution after dynamic culture, indicating that the platform supports continuous observation of barrier formation and damage progression. Compared with the fixed BBB chips reported in recent years [9,10], this modular structure gives up some integration in exchange for free combination of the injury-induction, drug-dosing, and sampling workflows, which better suits the two-stage design of this study, namely gradient-based injury induction followed by gradient-based dosing. From a pharmacological evaluation perspective, the methodological contribution of this platform stems from its spatiotemporally resolved multi-marker readout strategy. Each concentration-defined region generates parallel samples at successive time points for off-chip molecular assays including effluent ATP, cytokines, RT-qPCR, and transcriptomics, coupled with fluorescence imaging of ROS, NO, ICAM-1, and ZO-1. This design enables independent resolution of the temporal sequence of injury and intervention for each concentration region within a single platform.

The choice of pressure-sensitive adhesive for module integration, rather than plasma bonding or mechanical clamping, follows directly from the reversibility requirement. Plasma bonding is irreversible and would prevent the on-demand assembly and separation on which the stepwise workflow depends. Metal or magnetic clamping is reversible but adds footprint and dead volume, concentrates mechanical stress at the clamp edges, and provides no registration accuracy by itself, whereas the alignment micropillars fix the two modules in place before the adhesive is applied. Pressure-sensitive adhesive therefore offers the only option that is simultaneously reversible, self-registering with the micropillars, and compatible with the flat module interface.

The operating conditions likewise reflect a deliberate priority between two physical requirements. Both questions addressed here, namely how endothelial inflammatory activation and injury evolve with stimulus concentration and how compound efficacy varies with dose, are concentration-resolved by nature, and the platform was therefore optimized for gradient fidelity. As calculated in Section 4.1, a physiological wall shear stress and a stable Christmas-tree gradient cannot be achieved simultaneously within one module, because the flow rates required for the former preclude the diffusive mixing on which the latter depends [3]. The detachable format nevertheless offers a way to combine the two in sequence: cells may be shear-conditioned in the standalone culture module under high flow, with the gradient module mounted afterwards for exposure, a staged operation that we plan to implement in future work.

Two established assay formats deserve explicit comparison. Multiwell formats, including the ubiquitous 96-well plate, offer far higher sample throughput than the present four-channel device and remain the appropriate choice for large endpoint screens; however, each well holds a single, uniform concentration under static conditions, so a spatial concentration gradient cannot be imposed on a shared cell population, and perfusion is absent. Electric cell–substrate impedance sensing (ECIS) provides label-free, real-time monitoring of barrier integrity [20] and would complement the present optical and biochemical readouts; integrating impedance electrodes into the detachable modules is a natural direction for the next iteration of the platform, as noted in the limitations. The platform presented here is therefore complementary to, rather than a replacement for, these established formats: it trades sample throughput for concentration resolution and stepwise operability.

The gradient injury-induction results resolve a temporal order of inflammatory barrier damage: extracellular ATP and IL-8 rose significantly as early as 8 h of exposure, the earliest of all readouts; ROS and NO increased significantly at 12 h; IL-1β and IL-6 followed from 16 h onward; and structural damage to ZO-1 appeared last and worsened with time. This order matches the pathological logic in which danger signals and rapid chemokine responses act first, oxidative stress and the broader cytokine program are amplified next, and tight junctions finally disintegrate. As a classic damage-associated molecular pattern, ATP released from injured cells can activate the NLRP3 inflammasome via the P2X7 receptor and thereby amplify IL-1β and other pro-inflammatory signals [21], and recent studies have also listed it as one of the drivers of BBB disruption after ischemic stroke [22].

The transcriptomic results support the above model at the systems level. The number of differentially expressed genes increased continuously with the TNF-α concentration, with down-regulated genes accounting for the larger proportion, suggesting that high-intensity inflammatory stimulation may act mainly through repressive remodeling of the endothelial transcriptional program. The ten hub genes are almost all members of the interleukin and chemokine families, and the KEGG-enriched TNF, IL-17, and NF-κB pathways are precisely the canonical downstream pathways of TNF-α [23], indicating that the transcriptional response on the chip did not deviate from the known inflammatory signaling framework. *CXCL10*, the only hub gene down-regulated along the gradient, is driven mainly by interferons rather than by TNF-α [24], and its down-regulation provides indirect confirmation that this model operates in a TNFR-axis-dominated mode of stimulation. Dual validation by RT-qPCR and by ICAM-1 immunofluorescence at the mRNA and protein levels guarantees the reliability of the hub network as the basis for subsequent target screening.

Within the hub network, *ICAM1* is distinctive in that it is the only member located on the endothelial cell surface that performs effector function directly. Secreted cytokines act through microenvironmental diffusion and receptor relay, whereas ICAM-1, once clustered on the membrane surface, directly determines whether leukocytes can anchor to the endothelium. Upstream, the binding of TNF-α to TNFR1 recruits an adaptor protein complex that activates IKK and promotes IκBα degradation; the released NF-κB p65/p50 dimer enters the nucleus and binds the κB site in the *ICAM1* promoter region to drive transcription, with AP-1 and other factors acting synergistically [23,25,26]. DEX suppresses the induced expression of *ICAM1* precisely by interfering with the binding of NF-κB to this promoter [26], which corroborates our finding that DEX down-regulated ICAM-1 at both the mRNA and protein levels. Downstream, Glu34 in the D1 domain of the ICAM-1 extracellular region coordinates with the metal-ion-dependent adhesion site of the leukocyte integrin LFA-1, constituting the molecular basis of firm leukocyte adhesion [27,28]; Asp229/Glu254 in D3 bind Mac-1 and participate in transendothelial migration [29]; and D3–D4-mediated homodimerization increases ligand-binding avidity [27,30]. In brain microvascular endothelial cells, ICAM-1 clustering can also trigger intracellular signaling and actin cytoskeleton rearrangement, promoting lymphocyte transendothelial migration and weakening junctional integrity [28,29], and is a central link in leukocyte infiltration of the BBB under inflammatory conditions [31,32]. In this study, ICAM-1 was up-regulated in step with the TNF-α gradient at both the mRNA and protein levels and appeared together with ZO-1 damage, indicating that the chip model recapitulates the key features of the in vivo process described above. Within this framework, the direct binding of the two flavonoids to ICAM-1 has clear mechanistic implications. Their binding region lies near D2–D3 of the extracellular region. Although this is not the D1 interface that LFA-1 engages directly, D2 maintains the structural integrity of the D1 binding site [33], and D3 carries the Mac-1 binding site [27], so stable occupation of this region by a small molecule could in principle weaken the interaction between ICAM-1 and the integrins in an allosteric manner. The dynamics simulations further showed that the QUER complex maintained more hydrogen bonds and smaller conformational fluctuations throughout, whereas the KAE complex entered dynamic equilibrium only after pronounced conformational rearrangement. The only structural difference between the two is the hydroxyl group at the 3′ position of the B ring, which provides QUER with an additional hydrogen-bond donor and explains its binding and stability advantages at the atomic level.

The gradient dosing results are generally consistent with the above mechanistic predictions. DEX produced the largest inhibition across all readouts, consistent with its position as a first-line clinical anti-inflammatory drug, and this also verified the pharmacodynamic sensitivity of the model [34]. The 20 μM top concentration of DEX defines this positive-control reference for assay sensitivity and does not emulate clinical plasma exposure, so the comparison with QUER and KAE is pharmacodynamic rather than a simulation of clinical dosing. The prominent feature of QUER is its earlier onset of action: at 20 μM, extracellular ATP had decreased significantly by 8 h after dosing; at low concentrations, ROS was significantly reduced by 12 h; and significant inhibition of IL-8 secretion also appeared earlier than with DEX. Given that ROS and ATP lie upstream of the inflammatory cascade [7], this temporal characteristic suggests that the value of QUER may lie not in the strength of end-stage inhibition but in timely intervention against early injury events. By 48 h, QUER inhibited ROS and ICAM-1 significantly more strongly than KAE and delayed ZO-1 damage to an extent comparable to DEX; RT-qPCR showed that its suppression of *ICAM1* mRNA was likewise significantly stronger than that of KAE, corroborating the findings at the protein level. The efficacy difference between QUER and KAE is consistent in direction across four levels, namely binding energy, dynamic stability, mRNA, and protein, forming the most complete structure–activity evidence chain in this study, and it is also consistent with reports of the multi-target protective effects of QUER in cerebrovascular and neuroinflammatory models [35,36]. In addition, the broad down-regulation of the five hub-gene mRNAs by both flavonoids indicates that their actions are not limited to ICAM-1. This multi-target character is both an advantage of flavonoid ingredients and a reason why their in vivo effects are difficult to attribute to a single target [37]. A further detail of Figure 7 deserves comment: cytokine and ATP levels in the injury control (0 μM) continued to rise over the 48 h of the experiment. This increase is expected from the experimental design: in the drug-intervention experiment all four lines were perfused with 30 ng/mL TNF-α throughout, because inlet A received medium containing 30 ng/mL TNF-α alone and inlet B received medium containing 30 ng/mL TNF-α plus 20 μM compound (Section 4.5), so the 0 μM line is a continuously injured control whose biomarkers are expected to follow the pathological trend. The rise therefore reflects sustained TNF-α-driven injury and cumulative secretion into the collected effluent rather than continued cell proliferation—the cells had already reached confluence by 12 h of static culture (Figure 1D)—and the same monotonic increase was observed in the gradient modeling experiment (Figure 2A–D), where it captures the time course of inflammatory activation itself.

This study has several limitations. The most direct limitation comes from the model itself: the chip uses only the single hCMEC/D3 cell line and does not incorporate neurovascular unit components such as astrocytes, pericytes, and microglia; leukocytes were not included, so adhesion and migration events could not be observed directly; and a single TNF-α stimulus was used without in vivo validation, so extrapolation of the conclusions to complex neuroinflammatory situations should be made with caution. Even within the scope of the model, the direct binding of the two flavonoids to ICAM-1 and the allosteric regulation are currently supported only by molecular docking and dynamics simulations; they still need to be confirmed by surface plasmon resonance, microscale thermophoresis, or cellular thermal shift assays, and their functional consequences need to be examined in leukocyte–endothelial adhesion experiments. Moving from a candidate ingredient toward application also means confronting the generally low solubility and oral bioavailability of flavonoids [38]; this study did not examine their exposure levels across the BBB either, and the bias of TCMSP screening toward database-recorded ingredients likewise remains to be addressed in subsequent work. To this end, future studies can introduce multi-cell co-cultures and primary cells, add molecular-binding and adhesion-function experiments, and verify the brain exposure and anti-inflammatory effects of QUER in animal models, advancing both the platform and the candidate ingredient toward application. In addition, the current open-channel, detachable format is not compatible with integrated electrodes, so TEER and apparent permeability could not be measured; the wall shear stress under gradient operation (0.02–0.03 dyn/cm^2^) is below the physiological range of brain microvessels by design, because the flow rates required for physiological shear are incompatible with gradient formation (Section 4.1); and small-molecule absorption into PDMS, together with the use of nominal rather than directly measured outlet concentrations, constitutes a further caveat. A methodological caveat concerns the RT-qPCR normalization: although *GAPDH* expression was stable across channels in the RNA-seq data (Section 4.6), this reference gene can respond to inflammatory stimulation in other systems [39,40], and confirmation of the qPCR trends with a second, independently validated reference gene would further strengthen the analysis. A staged configuration in which cells are shear-conditioned in the standalone culture module before gradient exposure, electrode-integrated modules, and direct outlet quantification defines the next iteration of the platform.

## 4. Materials and Methods

### 4.1. Chip Design

The modular chip consists of two parts (Figure 1): a cell culture module (CCM) and a liquid concentration gradient generator module (LCGGM). The CCM contains four independent channels (1.5 mm wide, 0.15 mm deep) for cell loading and substance delivery, and four alignment holes at the sides of the channel region allow rapid assembly of the two modules. The LCGGM follows a classical Christmas-tree design, with two inlets, four outlets, and four alignment micropillars. Both modules are built on ultra-thin, high-transparency glass substrates (Mi-G01, Jianmi, Wuhan, China). During assembly, the four outlets of the LCGGM are aligned with the four inlets of the CCM through the micropillars and joined by pressure-sensitive adhesive, which allows fast and accurate module integration.

The wall shear stress τ in the culture channels was estimated for a rectangular channel as τ = 6μQ/(w·h^2^), where μ is the medium viscosity (0.7–1.0 mPa·s at 37 °C), Q is the per-channel flow rate (1 μL/min, i.e., two inlets at 2 μL/min each divided among four channels), and w and h are the channel width (1.5 mm) and depth (0.15 mm). This gives τ ≈ 0.02–0.03 dyn/cm^2^ and a mean flow velocity of about 74 μm/s (Reynolds number ≈ 0.01, fully laminar). Because τ scales linearly with Q in this geometry, raising it toward the range considered physiological for brain microvascular endothelium [3] would require increasing the flow rate by two to three orders of magnitude; the residence time in the Christmas-tree mixing network would decrease by the same factor, falling well below that required for complete diffusive mixing, and the stepwise gradient could no longer be established. The operating flow rate was therefore selected to prioritize gradient fidelity, on which both research questions of this study depend, rather than shear conditioning.

### 4.2. Chip Fabrication

Both modules were fabricated by casting poly-dimethylsiloxane (PDMS; Sylgard 184, Dow, Midland, MI, USA) into aluminum molds (MesoBioSys Co., Ltd., Wuhan, China). After oxygen plasma treatment (60 s, 500 mTorr, 100 W; PlasmaEZ, Micro-blox Technologies (Beijing) Co., Ltd., Beijing, China), the PDMS replicas were irreversibly bonded to the glass substrates. Before assembly, pressure-sensitive adhesive was cut with a CO_2_ laser (VSL2.30, Universal Laser Systems, Scottsdale, AZ, USA) to match the outline of the LCGGM, leaving four holes at the alignment positions. The adhesive was attached to the bottom of the LCGGM under a clean bench and sterilized by ultraviolet irradiation for 2 h. Guided by the alignment micropillars, the LCGGM was then laminated onto the upper surface of the CCM to complete the chip. The key dimensions of both modules are labeled in the design drawing provided as Figure S1 of the Supplementary Materials. During operation, any leakage at the module interface is immediately apparent because the culture medium contains phenol red.

### 4.3. Functional Characterization of the Chip

Gradient formation was verified using sodium fluorescein as a tracer. A sodium fluorescein solution (20 μg/mL) and ultrapure water were perfused through the two inlets of the LCGGM at 2 μL/min with a syringe pump (JSP-01-4A, JianMi Co., Ltd., Wuhan, China). After stepwise mixing in the Christmas-tree network, effluent from each of the four outlets was collected every 12 h, and the fluorescence of all fractions was read on a microplate reader (SPARK, TECAN, Männedorf, Switzerland) at the end of the 48 h experiment.

### 4.4. Cell Loading and Culturing

The hCMEC/D3 human brain microvascular endothelial cell line [41] was purchased from Procell (CL-0843, Wuhan, China) and maintained in hCMEC/D3-specific medium (CM-0843, Procell, Wuhan, China). Before cell loading, the CCM was sterilized by ultraviolet irradiation for 2 h in a clean bench and rinsed sequentially with 75% ethanol and sterile water. To promote cell adhesion, the channels were filled with type I collagen (150 μg/mL; 354249, Corning, NY, USA) and incubated for 12 h. hCMEC/D3 cells were suspended at 5 × 10^6^ cells/mL and injected through the channel inlet at 50 μL/min with a syringe pump. The CCM was then transferred to a cell culture incubator (STERI-CYCLE, Thermo Scientific, Waltham, MA, USA). hCMEC/D3 cells were labeled with CellTrace^™^ Red CMTPX (40717ES60, Yeasen, Shanghai, China) and continuously observed throughout the 12 h static culture period.

### 4.5. Chip Operation, Inflammation Modeling, and Drug Intervention

After 12 h of static culture, the LCGGM was mounted onto the CCM through the alignment micropillars and fixed with pressure-sensitive adhesive to ensure sealing. Fresh medium was perfused through both inlets of the LCGGM at 2 μL/min for another 12 h of dynamic culture.

For inflammation modeling, blank medium and medium containing 30 ng/mL TNF-α (C600021-0010, BBI, Shanghai, China) were delivered to inlets A and B, respectively, and the exposure lasted 48 h. After mixing in the Christmas-tree network, a TNF-α gradient ranging from 0% to 100% (nominal concentrations of 0, 10, 20, and 30 ng/mL) was established across Lines 1 to 4. For drug intervention, inlet A received medium containing 30 ng/mL TNF-α alone, while inlet B received medium containing 30 ng/mL TNF-α plus 20 μM QUER (HY-18085, MCE, Monmouth Junction, NJ, USA), KAE (HY-14590, MCE, Monmouth Junction, NJ, USA), or DEX (HY-14648, MCE, Monmouth Junction, NJ, USA); a drug gradient ranging from 0 to 20 μM (nominal concentrations of 0, 6.67, 13.33, and 20 μM) was thereby established across Lines 1 to 4, with TNF-α held at 30 ng/mL in all channels. The compounds were thus administered concurrently with TNF-α from the onset of exposure (a co-treatment regimen).

### 4.6. Transcriptome Sequencing and RT-qPCR

Total RNA was extracted as described in our previous publication [42]. Library preparation and sequencing were performed by BGI (Shenzhen, China). Briefly, mRNA was enriched from total RNA using Oligo(dT)-attached magnetic beads, fragmented, and reverse transcribed into double-stranded cDNA; after end repair and adapter ligation, the ligation products were PCR-amplified, purified with the AMPure XP system (Beckman Coulter, Brea, CA, USA), and library quality was assessed on an Agilent 4150 Bioanalyzer (Agilent, Santa Clara, CA, USA). Qualified libraries were circularized and sequenced on the DNBSEQ platform (BGI, Shenzhen, China).

At 48 h after TNF-α exposure or drug intervention, cells were collected for RNA extraction and RT-qPCR analysis. Purified RNA was reverse transcribed with the Transcriptor First Strand cDNA Synthesis Kit (04896866001, Roche, Mannheim, Germany), and RT-qPCR was run on a LightCycler 96 instrument (Roche, Mannheim, Germany) using the FastStart Essential DNA Green Master Kit (06924204001, Roche, Mannheim, Germany) according to the manufacturer’s instructions. Primer sequences (Table 1) were synthesized by Sangon Biotech (Shanghai, China). The cycling program was 95 °C for 15 min, followed by 40 cycles of 95 °C for 30 s, 55 °C for 1 min, and 72 °C for 30 s. Gene expression was normalized to *GAPDH* and calculated by the 2^−ΔΔCt^ method. The suitability of *GAPDH* as the reference gene was checked against the RNA-seq data, in which *GAPDH* showed no significant differential expression between any treated line (Lines 2–4) and Line 1 (0 ng/mL) (|log2FC| < 0.5 and adjusted *p* > 0.05 in all pairwise comparisons), supporting its stability under TNF-α exposure in this system; reports that *GAPDH* can vary under inflammatory conditions in other systems [39] and recommendations for context-specific reference-gene validation [40] are nevertheless acknowledged in the limitations.

### 4.7. Bioinformatics Analysis

Differentially expressed genes (DEGs) were identified with DESeq2 on the Dr. TOM platform (BGI, Shenzhen, China), using |log2FC| > 0.5 and an adjusted *p*-value (*P*.adj) < 0.05 as thresholds. GO and KEGG enrichment analyses were carried out with the Phyper function in R to explore the major functions of the DEGs and the associated phenotypic changes. A protein–protein interaction (PPI) network was built from the STRING database (https://string-db.org/), and hub genes were identified by maximal clique centrality (MCC) topological analysis using the cytoHubba plugin in Cytoscape 3.7.1 (The Cytoscape Consortium, San Diego, CA, USA).

### 4.8. Network Pharmacology Analysis

*IL6*, *IL1B*, *CXCL8*, and *ICAM1* were used as query targets in the Traditional Chinese Medicine Systems Pharmacology Database and Analysis Platform (TCMSP, https://tcmsp-e.com/). Ingredients associated with each target were retrieved from the database, and the four ingredient lists were intersected to identify candidate compounds capable of acting on multiple targets simultaneously. The intersection nominated QUER, which was carried forward, together with its structural analog KAE, to molecular docking and molecular dynamics analyses.

### 4.9. Molecular Docking

The crystal structure of intercellular adhesion molecule-1 (ICAM-1) was retrieved from the PDB (entry 7BG7), and the 3D structures of QUER and KAE were downloaded from PubChem. The receptor and ligands were prepared in AutoDockTools 1.5.7 (The Scripps Research Institute, La Jolla, CA, USA), which included removing the HRV14 capsid protein chains and crystallographic water, adding polar hydrogens, assigning Gasteiger charges, and merging non-polar hydrogens. Semi-flexible docking was performed with AutoDock Vina 1.2.7 (The Scripps Research Institute, La Jolla, CA, USA).

### 4.10. Molecular Dynamics Simulation

The docking complexes of ICAM-1 with QUER or KAE were each simulated for 100 ns in GROMACS 2025. The protein was described by the AMBER14SB force field; ligand topologies were built with sobtop 1.0 (dev 3.1) using GAFF parameters, with charges assigned by the RESP method. Each system was solvated in a TIP3P water box, and Na^+^ and Cl^−^ ions were added to mimic physiological ionic conditions. After steepest-descent energy minimization, the system was equilibrated under NVT, and then NPT ensembles, followed by a 100 ns production run at 300 K and 1 bar under periodic boundary conditions. Trajectories were analyzed in GROMACS for RMSD, RMSF, SASA, and the number of protein–ligand hydrogen bonds, which together describe the stability, conformational drift, solvation, and persistence of intermolecular contacts of each complex.

### 4.11. Intracellular Reactive Oxygen Species and Nitric Oxide Measurement

Intracellular reactive oxygen species (ROS) and nitric oxide (NO) were detected by fluorescence probing within the culture channels, following a previous protocol with minor modifications [42,43]. The CM-H2DCFDA kit (C6827, ThermoFisher, Waltham, MA, USA) and the DAF-FM DA probe (S0019M, Beyotime, Shanghai, China) were used for ROS and NO, respectively. Working solutions were freshly prepared by diluting the stock solutions in Hank’s Balanced Salt Solution (HBSS) according to the manufacturers’ instructions. The CCM channels were first rinsed with HBSS, then loaded with the working solution and incubated for 20 min in the dark. After a final HBSS rinse to remove excess probe, the chip was imaged on a confocal microscope (SpinSR, Olympus, Tokyo, Japan). Measurements were taken at 12, 24, and 48 h.

### 4.12. Biomarker Analysis of Chip Effluent

Effluent was analyzed as described previously with minor modifications [42,44]. Once TNF-α exposure or drug intervention started, the chip was kept under continuous perfusion, and the effluent from each channel was collected separately at its own outlet every 8 h (at 8, 16, 24, 32, 40, and 48 h) until the end of the experiment, without pooling or dilution. Samples were stored at −80 °C immediately after collection. Pro-inflammatory cytokines were quantified with commercial sandwich ELISA kits (colorimetric readout at 450 nm): IL-8 (ab214030), IL-6 (ab46042), and IL-1β (ab100562; all from Abcam, Waltham, MA, USA); extracellular ATP was quantified with a luciferase-based luminescence assay (E299, Dojindo, Kumamoto, Japan). Concentrations were calculated from standard curves run on the same plate.

### 4.13. Immunofluorescence

Cells in the CCM were fixed with 4% paraformaldehyde (E672002, BBI, Shanghai, China) for 15 min at room temperature, then permeabilized and blocked for 20 min with immunostaining blocking buffer (P0260, Beyotime, Shanghai, China) containing 0.3% Triton X-100 (A110694, Sangon Biotech, Shanghai, China). Samples were incubated overnight at 4 °C with rabbit anti-human ZO-1 (ab221547, Abcam, USA) or rabbit anti-human ICAM-1 (ab282575, Abcam, Waltham, MA, USA) diluted in primary antibody dilution buffer (P0109, Beyotime, Shanghai, China), followed by an Alexa Fluor 488-conjugated goat anti-rabbit IgG H&L secondary antibody (ab150077, Abcam, Waltham, MA, USA). For the ZO-1 characterization of the intact monolayer (Figure 1E), nuclei were counterstained with 0.5 μg/mL DAPI (D9542, Sigma-Aldrich, St. Louis, MO, USA) prepared in PBS; for the subsequent fluorescence assays, cell context and viability were instead verified by bright-field inspection of the confluent monolayer and by the intracellular ATP measurements described above. Images were acquired on a confocal laser scanning microscope (SpinSR, Olympus, Tokyo, Japan) under identical acquisition settings within each experiment; for quantification, three randomly selected fields per channel per time point were imaged, and the mean fluorescence intensity was averaged over fields before statistical analysis.

### 4.14. Image and Statistical Analysis

Images were analyzed with ImageJ (1.54g version, National Institutes of Health, Bethesda, MD, USA), and statistics were computed in SPSS 22.0 (SPSS Inc., Chicago, IL, USA). Unless otherwise stated, n = 3 denotes three independently fabricated and independently operated chips (biological replicates, using different cell passages on different days), and data are expressed as mean ± standard deviation (SD). Normality and homogeneity of variance were checked with the Shapiro–Wilk and Levene tests, respectively. Time-course data from repeated effluent sampling (Figure 2 and Figure 7) were analyzed with two-way repeated-measures ANOVA (factors: time and concentration), with chip included as a random effect, and main effects and the time × concentration interaction were reported. Endpoint comparisons (RT-qPCR and immunofluorescence) used one-way ANOVA followed by Dunnett’s test against Line 1 (0 ng/mL) or the injury control (0 μM); Dunnett’s test was selected because every comparison is made against a single reference group, for which it controls the family-wise error rate with greater power than all-pairwise procedures such as Tukey’s test. *p* < 0.05 was considered statistically significant.

## 5. Conclusions

This study developed a detachable, modular BBB chip that reversibly integrates a Christmas-tree concentration gradient generator module with a cell culture module, and established on this platform a stable, reproducible TNF-α-gradient inflammation model, which resolved the temporal order of barrier injury from early extracellular ATP release and oxidative stress, through pro-inflammatory cytokine accumulation, to eventual structural disruption of ZO-1. Transcriptomic analysis pinpointed a ten-gene hub network dominated by interleukins and chemokines, with *ICAM1* as the key surface effector; network pharmacology, molecular docking, and 100 ns molecular dynamics simulations then converged on quercetin, predicted to bind ICAM-1 more favorably than its structural analog kaempferol. Gradient dosing confirmed this prediction: quercetin acted earlier than dexamethasone on upstream injury signals, showed stronger overall anti-inflammatory efficacy than kaempferol with consistent advantages at both the mRNA and protein levels, and delayed ZO-1 damage to an extent comparable with dexamethasone, forming a coherent structure–activity evidence chain from atomic binding to cellular function. Because the two flavonols differ only by the 3′-hydroxyl on the B ring, this chain also links a single structural feature to anti-inflammatory potency. The modular platform and the gradient-based screening strategy it supports are readily transferable to the screening and mechanistic analysis of other natural-product ingredients, and follow-up work with multicellular co-cultures and animal models will further test the translational value of both the platform and quercetin.

## Figures and Tables

**Table 1:** Primer sequences for RT-qPCR.

Gene	Sequence
*IL1B*	F: ATGATGGCTTATTACAGTGGCAA R: GTCGGAGATTCGTAGCTGGA
*IL1A*	F: TGGTAGTAGCAACCAACGGGA R: ACTTTGATTGAGGGCGTCATTC
*ICAM1*	F: ATGCCCAGACATCTGTGTCC R: GGGGTCTCTATGCCCAACAA
*IL6*	F: GAAGAGCGCCGCTGAGAAT R: GTGCAGAGGGTTTAATGTCAACT
*CXCL8*	F: ATGACTTCCAAGCTGGCCGTGG R: TCTCAGCCCTCTTCAAAAACTACTC
*GAPDH*	F: CCAGCCGAGCCACATCGCTC R: ATGAGCCCCAGCCTTCTCCAT

## Data Availability

The raw sequencing data generated in this study have been deposited in the NCBI Sequence Read Archive (SRA) under BioProject ID PRJNA1531742 (https://www.ncbi.nlm.nih.gov/bioproject/PRJNA1531742, accessed on 14 October 2026) and are publicly available. The key dimensions of both chip modules are provided in the Supplementary Materials (Figure S1). Further inquiries can be directed to the corresponding authors.

## Supplementary Materials

The following supporting information can be downloaded at: https://www.mdpi.com/article/10.3390/ph19101655/s1, Figure S1: Design drawing of the two chip modules with key dimensions; Figure S2: Representative fluorescence images of intracellular ROS and NO and ZO-1 immunofluorescence in the gradient inflammation model; Figure S3: Representative fluorescence images of intracellular ROS and NO under concentration-gradient treatment with DEX, QUER, or KAE; Figure S4: Representative immunofluorescence images of ICAM-1 and ZO-1 under concentration-gradient treatment with DEX, QUER, or KAE.

## Abbreviations

The following abbreviations are used in this manuscript:

AP-1	Activator protein-1
ATP	Adenosine triphosphate
BBB	Blood–brain barrier
CCM	Cell culture module
DAPI	4′,6-Diamidino-2-phenylindole
DEX	Dexamethasone
DEG	Differentially expressed gene
GAPDH	Glyceraldehyde-3-phosphate dehydrogenase
GO	Gene Ontology
HBSS	Hank’s Balanced Salt Solution
hCMEC/D3	Human cerebral microvascular endothelial cell line D3
ICAM-1	Intercellular adhesion molecule-1
KAE	Kaempferol
KEGG	Kyoto Encyclopedia of Genes and Genomes
LCGGM	Liquid concentration gradient generator module
LFA-1	Lymphocyte function-associated antigen-1
MCC	Maximal clique centrality
MD	Molecular dynamics
NF-κB	Nuclear factor-κB
NLRP3	NLR family pyrin domain containing 3
NO	Nitric oxide
PDMS	Polydimethylsiloxane
PPI	Protein–protein interaction
QUER	Quercetin
RMSD	Root-mean-square deviation
RMSF	Root-mean-square fluctuation
ROS	Reactive oxygen species
RT-qPCR	Reverse transcription quantitative polymerase chain reaction
SASA	Solvent-accessible surface area
TCMSP	Traditional Chinese Medicine Systems Pharmacology Database and Analysis Platform
TNFR1	Tumor necrosis factor receptor 1
ZO-1	Zonula occludens-1
