# Supplementary material for: Detachable Modular Blood–Brain Barrier-on-a-Chip with a Concentration Gradient Generator for Stepwise Inflammation Modeling and Drug Evaluation
Source: Pharmaceuticals (Basel). 2026 Oct 20;19(10):1655. doi: 10.3390/ph19101655 (PMC13708139; doi:10.3390/ph19101655)
Supplement: Supplementary file 1 [file pharmaceuticals-19-01655-s001.zip › pharmaceuticals-4593291-supplementary.pdf]

Detachable Modular Blood–Brain Barrier-on-a-Chip with a Concentration Gradient Generator for Stepwise Inflammation Modeling and Drug Evaluation

Supplementary Materials

Zezhi Li [1,2], Haizhu Meng [3], Xinmeng Liao [1], Shuqi Wang [2], Dan Hu [2], Lijuan Chen [4], Yuanhang Chang [5], Junwei Zhao [1], Xiang Li [1,*] and Jianping Xie [1,*]

1 Beijing Life Science Academy, Building 1, Courtyard 7, Yingcai South 1st Street, Changping District, Beijing 102209, P.R. China; lzzdyq1992@163.com (Z.L.); liaoxm@blsa.com.cn (Xinmeng Liao); zhaojw@blsa.com.cn (J.Z.)

2 Beijing Technology and Business University, No.11, Fucheng Road, Haidian District, Beijing 100048, P.R. China; wangshuqi@btbu.edu.cn (S.W.); hud@btbu.edu.cn (D.H.)

3 Shandong Medical and Pharmaceutical University, No. 318, Gangcheng East Street, Laishan District, Yantai 264003, P.R. China; mhz9683@163.com (H.M.)

4 Zhengzhou University of Light Industry, No. 136 Kexue Road, Zhengzhou 450001, P.R. China; chenlijuan5210@163.com (L.C.)

5 Qilu Medical University, No. 1678 Renmin Road, Zibo 255300, Shandong, P.R. China; changyuanhang@qlmu.edu.cn (Y.C.)

* Correspondence: ztridicp@126.com (J.X.); lixiang79ben@sina.com (X.L.)

Supplementary Figures

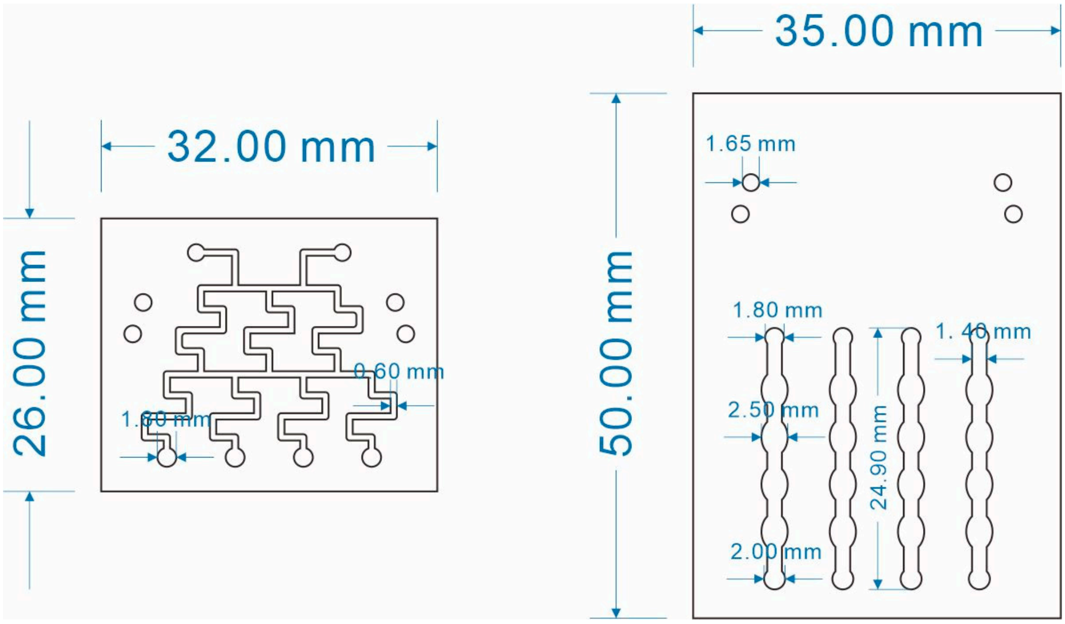

Figure S1. Design drawing of the two chip modules with key dimensions (units: mm). Left: concentration gradient generator module (Christmas-tree mixing network). Right: cell culture module comprising four parallel culture channels (Line 1–4). The complete set of design parameters and the files required to run the fabrication equipment are provided together with this Supplementary Materials file.

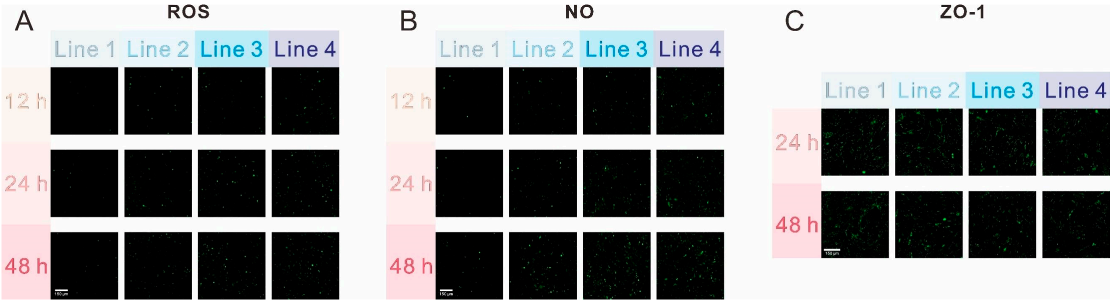

Figure S2. Representative fluorescence images validating the gradient inflammation model on the chip. Lines 1–4 received nominal TNF-α concentrations of 0, 10, 20, and 30 ng/mL, respectively (0%, 33%, 66%, and 100% of the inlet concentration). (A, B) Intracellular ROS (CM-H2DCFDA) and NO (DAF-FM DA) fluorescence in live cells at 12, 24, and 48 h of exposure. (C) ZO-1 immunofluorescence (green) at 24 and 48 h. Images are representative of three independent chips ($n = 3$); the corresponding quantification is shown in Figure 2E–G of the main text.

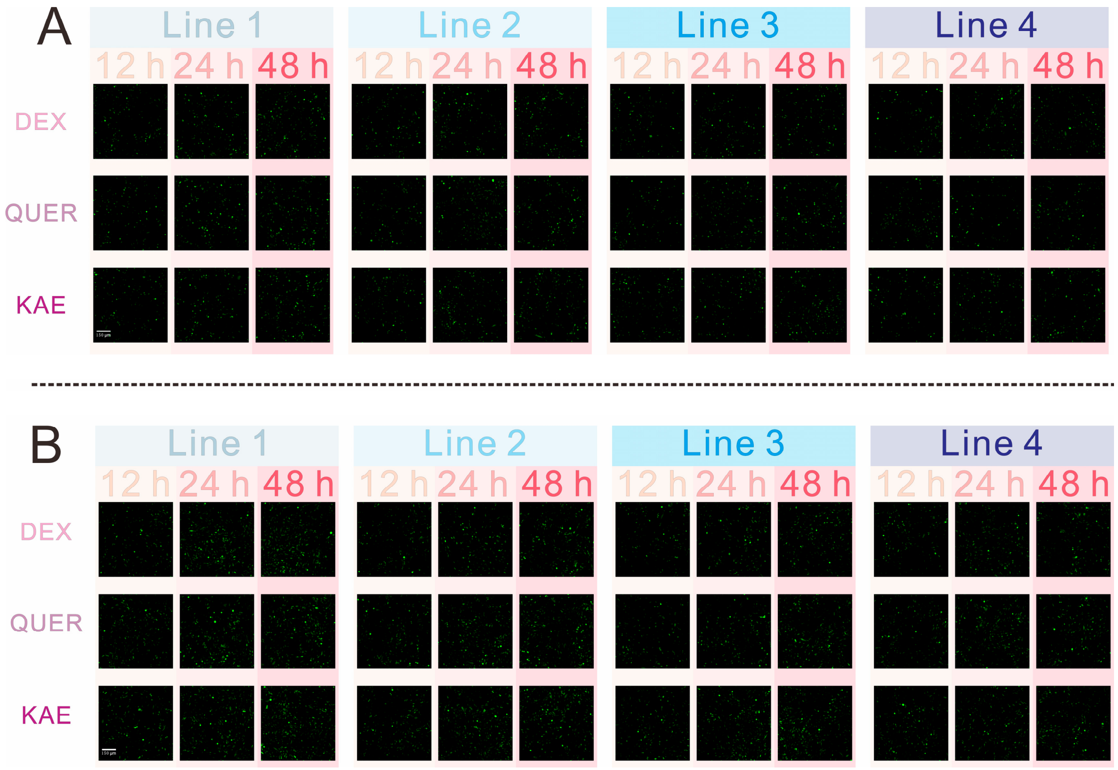

Figure S3. Representative fluorescence images of intracellular ROS (A) and NO (B) in live HCMEC/D3 cells treated with DEX, QUER, or KAE. Lines 1–4 received nominal drug concentrations of 0, 6.67, 13.33, and 20 μM, respectively, under constant 30 ng/mL TNF-α exposure, and images were acquired at 12, 24, and 48 h. Images are representative of three independent chips (n = 3); the corresponding quantification is shown in Figure 8 of the main text.

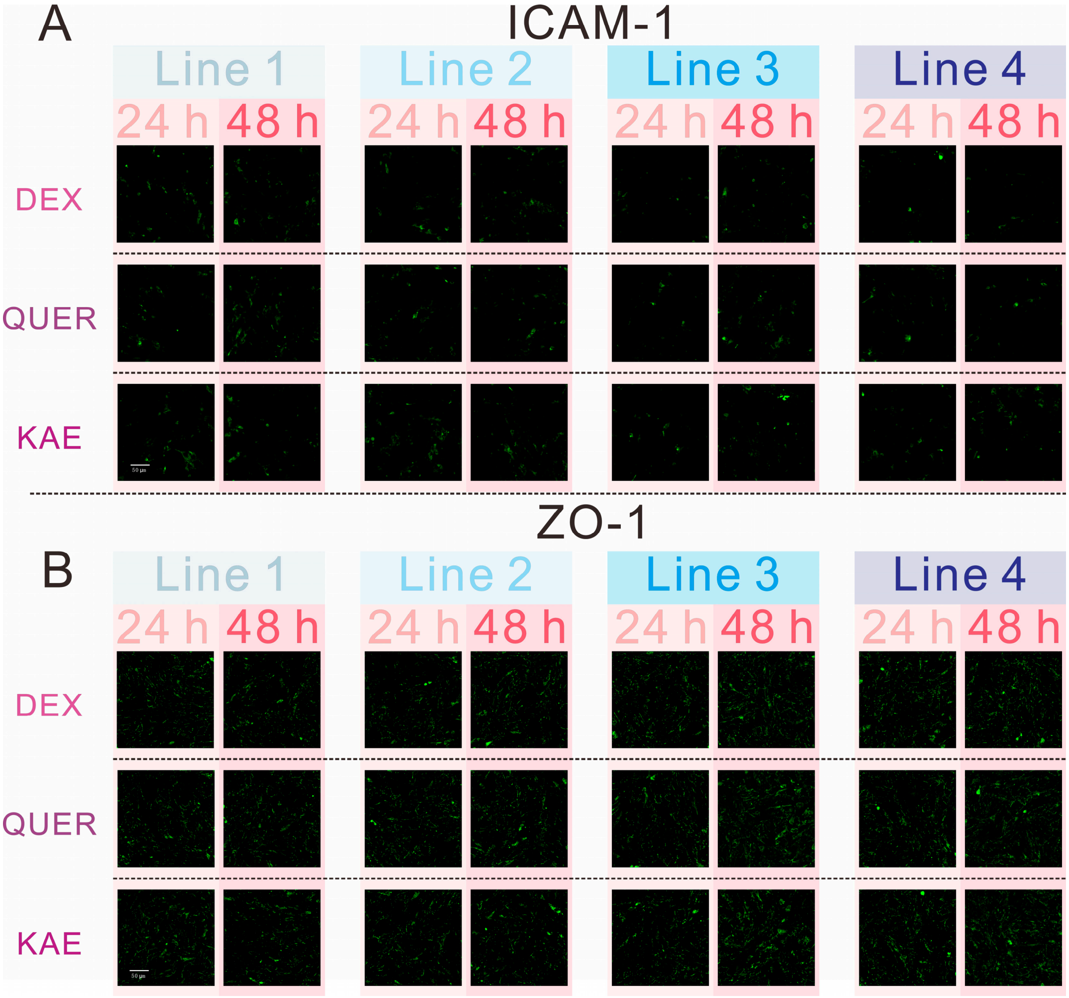

Figure S4. Representative immunofluorescence images of ICAM-1 (A) and ZO-1 (B; both green) in HCMEC/D3 cells treated with DEX, QUER, or KAE. Lines 1–4 received nominal drug concentrations of 0, 6.67, 13.33, and 20 μM, respectively, under constant 30 ng/mL TNF-α exposure, and images were acquired at 24 and 48 h. Images are representative of three independent chips (n = 3); the corresponding quantification is shown in Figure 9 of the main text.
